# COVID-19 Rapid Antigen Tests With Self-Collected vs Health Care Worker–Collected Nasal and Throat Swab Specimens: A Randomized Clinical Trial

**DOI:** 10.1001/jamanetworkopen.2023.44295

**Published:** 2023-12-06

**Authors:** Tobias Todsen, Kathrine K. Jakobsen, Mathias Peter Grønlund, Rasmus E. Callesen, Fredrik Folke, Helene Larsen, Annette Kjær Ersbøll, Thomas Benfield, Tobias Gredal, Mads Klokker, Nikolai Kirkby, Christian von Buchwald

**Affiliations:** 1 Department of Otorhinolaryngology, Head and Neck Surgery and Audiology, Copenhagen University Hospital, Rigshospitalet, Copenhagen, Denmark; 2 Copenhagen Academy for Medical Education and Simulation, Capital Region, Copenhagen, Denmark; 3 Department of Clinical Medicine, University of Copenhagen, Copenhagen, Denmark; 4 Copenhagen Emergency Medical Services, University of Copenhagen, Copenhagen, Denmark; 5 Department of Cardiology, Copenhagen University Hospital, Herlev Gentofte, Denmark; 6 Center for Diagnostics, Department of Health Technology, Technical University of Denmark, Kongens Lyngby, Denmark; 7 National Institute of Public Health, University of Southern Denmark, Copenhagen, Denmark; 8 Department of Infectious Diseases, Copenhagen University Hospital, Amager and Hvidovre, Hvidovre, Denmark; 9 Department of Clinical Microbiology, Rigshospitalet, Copenhagen University Hospital, Copenhagen, Denmark

## Abstract

**Importance:**

Self- or health care worker (HCW)–collected nasal swab specimens are the preferred sampling method to perform rapid antigen testing for COVID-19, but it is debated whether throat specimens can improve test sensitivity.

**Objective:**

To compare the diagnostic accuracy of self- and HCW-collected nasal vs throat swab specimens for COVID-19 rapid antigen testing.

**Design, Setting, and Participants:**

This per-protocol multicenter randomized clinical trial was conducted from February 15 through March 25, 2022. The participants, individuals aged 16 years or older requesting a COVID-19 test for diagnostic or screening purposes, had 4 specimens collected for individual testing at 1 of 2 urban COVID-19 outpatient test centers in Copenhagen, Denmark.

**Interventions:**

Participants were randomized 1:1 to self-collected or HCW-collected nasal and throat swab specimens for rapid antigen testing. Additional HCW-collected nasal and throat swab specimens for reverse transcriptase–polymerase chain reaction (RT-PCR) were used as the reference standard.

**Main Outcomes and Measures:**

The primary outcome was sensitivity to diagnose COVID-19 of a self- vs HCW-collected nasal and throat specimen for rapid antigen testing compared with RT-PCR.

**Results:**

Of 2941 participants enrolled, 2674 (90.9%) had complete test results and were included in the final analysis (1535 [57.4%] women; median age, 40 years [IQR, 28-55 years]); 1074 (40.2%) had COVID-19 symptoms, and 827 (30.9%) were positive for SARS-CoV-2 by RT-PCR. Health care worker–collected throat specimens had higher mean sensitivity than HCW-collected nasal specimens for rapid antigen testing (69.4% [95% CI, 65.1%-73.6%] vs 60.0% [95% CI, 55.4%-64.5%]). However, a subgroup analysis of symptomatic participants found that self-collected nasal specimens were more sensitive than self-collected throat specimens for rapid antigen testing (mean sensitivity, 71.5% [95% CI, 65.3%-77.6%] vs 58.0% [95% CI, 51.2%-64.7%]; *P* < .001). Combining nasal and throat specimens increased sensitivity for HCW- and self-collected specimens by 21.4 and 15.5 percentage points, respectively, compared with a single nasal specimen (both *P* < .001).

**Conclusions and Relevance:**

This randomized clinical trial found that a single HCW-collected throat specimen had higher sensitivity for rapid antigen testing for SARS-CoV-2 than a nasal specimen. In contrast, the self-collected nasal specimens had higher sensitivity than throat specimens for symptomatic participants. Adding a throat specimen to the standard practice of collecting a single nasal specimen could improve sensitivity for rapid antigen testing in health care and home-based settings.

**Trial Registration:**

ClinicalTrials.gov Identifier: NCT05209178

## Key Points

**Question:**

Do self-collected and health care worker (HCW)–collected throat swab specimens improve the accuracy of COVID-19 rapid antigen testing compared with nasal swab specimens?

**Findings:**

In this multicenter randomized clinical trial including 2941 participants (2674 with complete data), HCW-collected throat swabs were more sensitive than nasal specimens for rapid antigen testing; no difference was found between self-collected throat and nasal specimens. Adding throat to nasal specimens for individual rapid antigen testing increased sensitivity for HCW- and self-collected specimens.

**Meaning:**

HCW-collected throat specimens may have higher sensitivity than HCW-collected nasal specimens for COVID-19 rapid antigen testing, while the sensitivity of self-collected specimens may improve by combining nasal and throat specimens.

## Teaser

This randomized clinical trial compares the diagnostic accuracy of self-collected and health care worker–collected nasal and throat specimens for SARS-CoV-2 rapid antigen testing.

## Introduction

SARS-CoV-2 testing is crucial to provide early COVID-19 treatment for people at high risk of severe illness and to introduce measures that reduce transmission to others. Rapid antigen tests have been widely used during the COVID-19 pandemic due to low cost and the availability of over-the-counter tests, allowing early detection and self-isolation of infectious individuals at home.^1,2^ However, the rate of false-negative home-based rapid antigen test results has been widely debated during the Omicron variant surge, and it has been suggested that throat swab specimens could improve test sensitivity.^3^ Health authorities in the UK, Canada, and Israel recommend including a throat specimen for reverse transcriptase–polymerase chain reaction (RT-PCR) SARS-CoV-2 testing, while the US Food and Drug Administration (FDA) has only authorized rapid antigen tests for use with nasal specimens and advises against using throat specimens.^4^ Research with molecular SARS-CoV-2 testing suggests that throat specimens play an important role in the initial infection,^5^ while studies with rapid antigen tests are contradictive and range from advising against^6,7,8^ to recommending^9,10^ the use of throat specimens. Randomized clinical trials with head-to-head comparison of throat and nasal specimens are therefore needed.

## Methods

### Study Design

This investigator-initiated, multicenter randomized clinical trial (NCT05209178) was conducted at 2 public COVID-19 test centers in Copenhagen, Denmark, from February 15 until March 25, 2022. The participants had 4 specimens collected: 2 health care worker (HCW)–collected nasal and throat swab specimens for RT-PCR testing (reference standard) and, afterward, 2 nasal and throat swab specimens (randomized to either self- or HCW-collected) for rapid antigen testing. The trial protocol and statistical analysis plan are given in Supplement 1. The trial was reported to the Regional Ethics Committee of the Capital Region of Denmark and was considered exempt from further processing due to the small study intervention adding some extra samples (eAppendix 6 in Supplement 2). Participants provided verbal and written informed consent before enrollment. The study followed the Consolidated Standards of Reporting Trials (CONSORT) reporting guideline for reporting of data.

A computer-generated block randomization list was made by K.K.J. using an online randomization tool.^11^ The list was integrated into REDCap and used at the test centers to reveal whether the participant would have either self-collected (intervention group) or HCW-collected (control group) nasal and throat specimens for rapid antigen testing (eFigure 1 in Supplement 2).

### Participants

Danish citizens aged 16 years or older who requested a SARS-CoV-2 RT-PCR test at Testcenter Kastrup (Copehagen Airport) or Testcenter Valby in Copenhagen, Denmark, were invited to participate in the study. SARS-CoV-2 testing was free without the need for a referral during the pandemic in Denmark.^12,13^ Individuals with nasopharyngeal or oropharyngeal anomalies (eg, participants with a tracheostomy) were excluded from the study. After enrollment, participants provided information regarding the presence, type, and duration of symptoms and COVID-19 vaccination status (eAppendix 1 in Supplement 2).

### Molecular and Rapid Antigen Testing for SARS-CoV-2

The participants first had throat and nasal specimens collected by an HCW using a separate Meditec specimen collection swab with a nylon-flocked tip (Meditec A/S) for each location. The nasal swab was inserted about 4 cm in both nostrils or until resistance was met and rotated 3 times (eAppendix 2 in Supplement 2). The throat swab collected a specimen from the posterior oropharyngeal wall and palatine tonsils, avoiding the tongue and cheeks.^14,15^ The throat and nose swabs were placed in separate inactivation transport media (Wuxi NEST Biotechnology Co, Ltd) and stored at 4 °C until transported to the laboratory for separate RT-PCR testing (reference standard). The RT-PCR analyses were performed at the Technical University of Denmark (DTU), Lyngby, or at the Department of Clinical Microbiology, Rigshospitalet (eAppendix 3 in Supplement 2). An RT-PCR test was considered positive for SARS-CoV-2 if the cycling threshold (Ct) was 34 or lower for at least 1 of 2 target genes (a lower value indicates a higher viral load). If possible, the positive samples were also spike gene sequenced to identify SARS-CoV-2 variants of concern (eAppendix 4 in Supplement 2).

After the specimens were collected for RT-PCR testing, the participants were randomized 1:1 to either self-collected (intervention group) or HCW-collected (control group) nasal and throat specimens for rapid antigen testing. Flocked nasal swabs from a rapid antigen test kit (Standard Q COVID-19 Ag test, SD; Biosensor Inc) were used for both nasal and throat specimen collection for rapid antigen testing in the intervention and control groups. The participants in the intervention group were provided with instructional material consisting of a handout with written and visual steps for self-swabbing (eAppendix 2 in Supplement 2). A mirror was provided to the participants to guide the self-swabbing of the throat, including the posterior wall of the pharynx and the palatine tonsils.^15,16^ Each swab specimen was diluted in individual extraction buffers, and the extracted specimens were added to the well of separate lateral-flow test devices (Standard Q COVID-19 Ag test) according to the manufacturer’s instructions. The participants then called for an HCW at the test center, who collected the antigen test device and read the results as either positive, negative, or inconclusive after 15 to 30 minutes. The participants in the control group instead had nasal and throat swabbing performed by an HCW for rapid antigen testing who used the same swab technique as described for RT-PCR testing. The HCW then diluted the swab specimens in separate extraction buffers and analyzed the specimens with the same procedures as for the intervention group.

### Sample Size Justification

The power calculation was based on an expected 10% difference in the rate of SARS-CoV-2 detection for self-collected vs HCW-collected specimens^17^ and a prevalence of SARS-CoV-2 infection of 30%. We used the paired, 1-sided test in the pwr package in R, version 3.6.1 (R Project for Statistical Computing) to estimate that a sample of 2794 participants would give the trial 80% power at a 5% significance level.

### Statistical Analysis

A study participant was considered positive for SARS-CoV-2 if the pathogen was detected by RT-PCR (reference standard) in the nasal, throat, or both specimens. The reference standard was then used to calculate the sensitivity, specificity, positive predictive value, and negative predictive value of the rapid antigen testing of different specimens (eAppendix 5 in Supplement 2). The diagnostic accuracy of combined nasal and throat specimens for rapid antigen testing was estimated by adding the test results from each site, meaning the result from the combined approach was considered positive if the nasal, throat, or both specimens were positive. Participants with missing or invalid test results by either RT-PCR or rapid antigen testing were excluded. A logistic regression analysis was used to test differences in the proportions of true SARS-CoV-2 detection between specimens and between collection methods. To compare the proportions of true SARS-CoV-2 detection between specimens, a generalized estimating equations approach was used to account for repeated sampling within a participant. The test center was included as a fixed effect in both analyses. The Ct values from samples with positive RT-PCR results were compared using a general linear mixed model with a random effect of participants. The Ct values were logarithmic transformed to account for a skewed distribution. Values are presented as medians and IQRs or means with 95% CIs. We defined the level of statistical significance as 2-sided *P* < .05. Data were analyzed using SAS, version 9.4 (SAS Institute Inc).

We performed a post hoc analysis to explore the robustness of our findings when including invalid or missing rapid antigen tests as negative using an intention-to-treat approach.^18,19^ Furthermore, post hoc subgroup analyses of rapid antigen test results for symptomatic and asymptomatic participants were performed. To standardize the molecular reference test, a subgroup of participants with both nasal and throat specimens tested with RT-PCR at the same laboratory at DTU was conducted separately.

## Results

**Figure 1.**
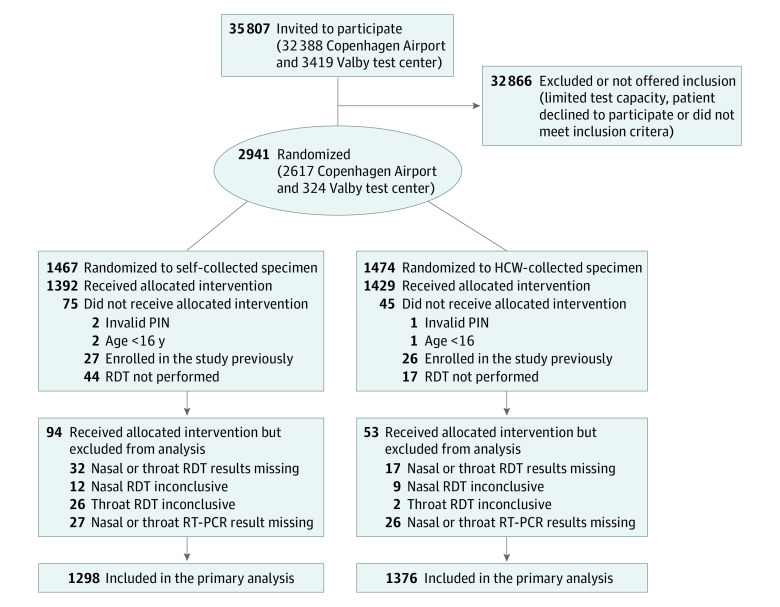
CONSORT Flow Diagram HCW indicates health care worker; PIN, social security number; RDT, rapid diagnostic antigen test; and RT-PCR, reverse transcriptase–polymerase chain reaction.

During the study period, 35 807 individuals requested a test in the test centers, and 2941 (8.2%) were enrolled in the study; 1271 (43.2%) were men, 1670 (56.8%) were women, the median age was 40 years (IQR, 28-55 years), and 2733 (92.9%) were vaccinated against COVID-19. The reasons for testing were COVID-19–like symptoms (1166 participants [39.6%]), exposure to infection (919 [31.2%]), screening (706 [24.0%]), and other (150 [5.1%]). Of all adults enrolled, 267 test cases (9.1%) were excluded from the analysis primarily due to prior enrollment or 1 or more inconclusive or missing test results. A total of 2674 participants (90.9%) had complete results for all RT-PCR and rapid antigen tests and were included in the final analysis (Table 1, Figure 1, and eTable 1 in Supplement 2). Of the 2674 participants (1139 [42.9% men]; 1535 [57.4%] women; median age, 40 years [IQR, 28-55 years]), 1074 (40.2%) had COVID-19 symptoms and 827 (30.9%) were positive for SARS-CoV-2 by RT-PCR (reference standard for a true-positive result) from throat (111 [4.2%]) or nasal (48 [1.8%]) specimens or both (668 [25.0%]) (eTable 2 in Supplement 2). The detection rate was 86.6% (95% CI, 84.3%-88.9%) for a nasal specimen and 94.2% (95% CI, 92.6%-95.8%) for a throat specimen. The median Ct value was lower for an HCW-collected nasal specimen than for an HCW-collected throat specimen (16.7 [IQR, 13.8-21.7] vs 22.8 [IQR, 20.2-25.7]; *P* < .001). Of 286 samples sequenced for the SARS-CoV-2 variants of concern, the majority were either Omicron BA.2 (191 [66.8%]) or BA.2.9 (75 [26.2%]) (eTable 3 in Supplement 2).

**Table 1.** Demographic and Clinical Characteristics of Participants Included in the Final Analysis

Characteristic	Participants, No. (%)		
	All (N = 2674)	Self-collected RDT (n = 1298)	HCW-collected RDT (n = 1376)
Sex			
Female	1535 (57.4)	777 (59.9)	758 (55.1)
Male	1139 (42.6)	521 (40.1)	618 (44.9)
Age, median (IQR), y	40 (28-55)	39 (28-55)	40 (28-55)
Symptoms present			
Yes	1074 (40.2)	535 (41.2)	539 (39.2)
No	1600 (59.8)	763 (58.8)	837 (60.8)
Vaccination status			
Vaccinated	2484 (94.2)	1213 (94.5)	1271 (94.0)
Nonvaccinated	152 (5.8)	71 (5.5)	81 (6.0)
Unknown	38 (1.4)	14 (1.1)	24 (1.7)
Reason for testing			
COVID-19–like symptoms	1074 (40.2)	535 (41.2)	539 (39.2)
Contact with infected person	833 (31.2)	398 (30.7)	435 (31.6)
Regular testing	633 (23.7)	312 (24.0)	321 (23.3)
Other	134 (5.0)	53 (4.1)	81 (5.9)
Time since first symptoms, No./total No. (%)^a^			
1 d	379/1071 (35.4)	188/533 (35.3)	191/538 (35.5)
2-3 d	527/1071 (49.2)	258/533 (48.4)	269/538 (50.0)
4-6 d	165/1071 (15.4)	87/533 (16.3)	78/538 (14.5)
Missing	3	2	1
Test center			
Copenhagen Airport	2369 (88.6)	1153 (88.8)	1216 (88.4)
Valby	305 (11.4)	145 (11.2)	160 (11.6)
RT-PCR test result, pooled			
Positive	827 (30.9)	380 (29.3)	447 (32.5)
Negative	1847 (69.1)	918 (70.7)	929 (67.5)
RDT result			
Nose			
Positive	495 (18.5)	224 (17.3)	271 (19.7)
Negative	2179 (81.5)	1074 (82.7)	1105 (80.3)
Throat			
Positive	520 (19.5)	208 (16.0)	312 (22.7)
Negative	2154 (80.5)	1090 (84.0)	1064 (77.3)

Abbreviations: HCW, health care worker; RDT, rapid diagnostic antigen test; RT-PCR, reverse transcriptase–polymerase chain reaction.

^a^ Among individuals with symptoms.

**Figure 2.**
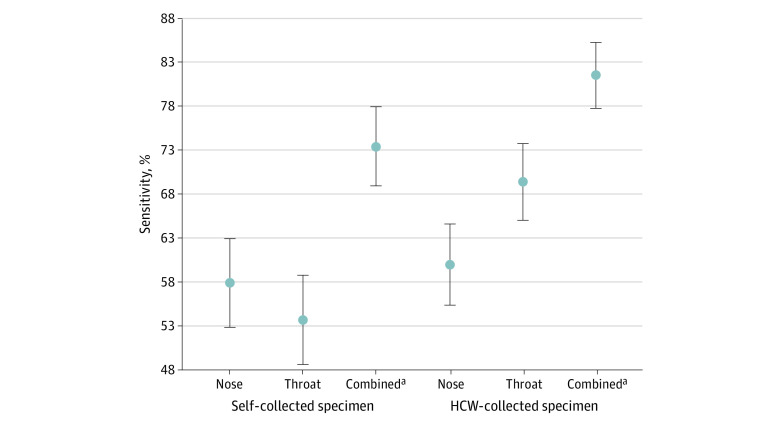
Sensitivity of Self-Collected and Health Care Worker (HCW)–Collected Nasal and Throat Specimens for Rapid Antigen Testing Error bars indicate 95% CIs. ^a^The rapid antigen test results from the nasal and throat specimens were summed to estimate the sensitivity of a combination of both specimens.

**Figure 3.**
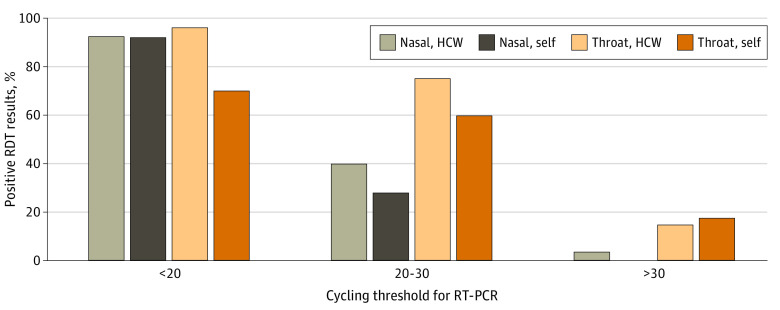
Percentages of Positive Rapid Diagnostic Antigen Test (RDT) Results for Self-Collected and Health Care Worker (HCW)–Collected Nasal and Throat Specimens by Reverse Transcriptase–Polymerase Chain Reaction (RT-PCR) Cycling Threshold Value

For a single specimen, the mean sensitivity for rapid antigen testing was lower for a self-collected throat specimen than for an HCW-collected throat specimen (53.7% [95% CI, 48.7%-58.7%] vs 69.4% [95% CI, 65.1%-73.6%]; *P* < .001) and was comparable for self-collected and HCW-collected nasal specimens (57.9% [95% CI, 52.9%-62.9%] vs 60.0% [95% CI, 55.4%-64.5%]; *P* = .53) (Figure 2 and eTable 4 in Supplement 2). The mean sensitivity was higher for HCW-collected throat specimens vs nasal specimens (69.4% [95% CI, 65.1%-73.6%] vs 60.0% [55.4%-64.5%]; *P* < .001), while no difference was found between self-collected throat specimens vs nasal specimens for rapid antigen testing (53.7% [95% CI, 48.7%-58.7%] vs 57.9% [95% CI, 52.9%-62.9%]; *P* = .17). In contrast, a subgroup analysis of participants with symptoms revealed that self-collected nasal specimens had significantly higher mean sensitivity than self-collected throat specimens (71.5% [95% CI, 65.3%-77.6%] vs 58.0% [95% CI, 51.2%-64.7%]; *P* < .001) (Table 2 and eFigure 4 and eTable 7 in Supplement 2). This was also confirmed in a subgroup analysis of participants with high SARS-CoV-2 load (Ct <20), in which we found lower sensitivity for self-collected throat specimens; self-collected nasal, HCW-collected nasal, and HCW-collected throat specimens had comparable sensitivity for rapid antigen testing (Figure 3 and eFigure 5 in Supplement 2). However, HCW-collected nasal specimens still had significantly lower mean sensitivity than HCW-collected throat specimens for rapid antigen testing (68.5% [95% CI, 62.8%-74.2%] vs 76.4% [95% CI, 71.2%-81.6%]; *P* = .03). The mean sensitivity of the combined nasal and throat specimens was higher for HCW-collected specimens vs self-collected specimens for rapid antigen testing (81.4% [95% CI, 77.8%-85.0%] vs 73.4% [95% CI, 69.0%-77.9%]; *P* = .006). A test strategy with combined nasal and throat specimens collected and analyzed on separate rapid antigen test devices significantly increased sensitivity for HCW-collected and self-collected specimens by 21.4 and 15.5 percentage points, respectively, compared with standard practice with a single nasal specimen (both *P* < .001). The specificity was greater than 99.5% for rapid antigen testing of all sample types, without any significant difference (Table 2 and eFigure 2 in Supplement 2). No adverse events were observed during the HCW-collected or self-collected nasal and throat swabs performed in the test centers.

**Table 2.** Diagnostic Accuracy of RDT of Enrolled Participants Based on Randomization and Subgroup Analysis of Symptomatic Participants^a^

Metric	RDT accuracy, % (95% CI)					
	Self-collected (n = 1298)			HCW-collected (n = 1376)		
	Nose	Throat	Nose and throat	Nose	Throat	Nose and throat
**All participants (N = 2674)**						
Sensitivity	57.9 (52.9-62.9)	53.7 (48.7-58.7)	73.4 (69.0-77.9)	60.0 (55.4-64.5)	69.4 (65.1-73.6)	81.4 (77.8-85.0)
Specificity	99.6 (99.1-100)	99.6 (99.1-100)	99.4 (98.8-99.9)	99.7 (99.3-100)	99.8 (99.5-100)	99.5 (99.0-99.9)
PPV	98.2 (96.5-100)	98.1 (96.2-99.9)	97.9 (96.2-99.6)	98.9 (97.7-100)	99.4 (98.5-100)	98.6 (97.5-99.8)
NPV	85.1 (83.0-87.2)	83.9 (81.7-86.0)	90.0 (88.2-91.9)	83.8 (81.6-86.0)	87.1 (85.1-89.1)	91.8 (90.1-93.5)
**Participants with symptoms (n = 1074)**						
Sensitivity	71.5 (65.3-77.6)	58.0 (51.2-64.7)	81.2 (75.8-86.5)	68.5 (62.8-74.2)	76.4 (71.2-81.6)	88.6 (84.7-92.5)
Specificity	99.4 (98.5-100)	99.1 (98.1-100)	98.8 (97.6-100)	99.3 (98.3-100)	99.6 (99.0-100)	98.9 (97.8-100)
PPV	98.7 (96.8-100)	97.6 (94.8-100)	97.7 (95.4-99.9)	98.9 (97.3-100)	99.5 (98.5-100)	98.7 (97.2-100)
NPV	84.7 (81.1-88.3)	78.9 (74.9-82.8)	89.3 (86.1-92.4)	78.0 (73.7-82.2)	82.6 (78.5-86.6)	90.7 (87.4-93.9)

Abbreviations: HCW, health care worker; NPV, negative predictive value; PPV, positive predictive value; RDT, rapid diagnostic antigen testing.

^a^ Missing and invalid RDTs were excluded from these calculations.

An intention-to-treat post hoc analysis confirmed that the mean sensitivity was higher for HCW-collected throat specimens than for HCW-collected nasal specimens for rapid antigen testing (69.8% [95% CI, 65.5%-74.0%] vs 59.9% [95% CI, 55.4%-64.4%]; *P* < .001) (eFigure 3 and eTable 5 in Supplement 2). Results of a subgroup analysis of participants with all RT-PCR tests performed in the same laboratory at DTU are provided in eTable 6 in Supplement 2.

## Discussion

This randomized clinical trial found that the sensitivity of SARS-CoV-2 rapid antigen testing was significantly higher for HCW-collected throat specimens compared with HCW-collected nasal specimens. In contrast, the self-collected nasal specimens had higher sensitivity than the self-collected throat specimens for symptomatic participants. Adding a throat swab to the standard practice of a single nasal swab may improve sensitivity for HCW-collected and self-collected specimens for rapid antigen testing.

To our knowledge, our study is the first to demonstrate that HCW-collected throat specimens for COVID-19 rapid antigen testing have a significantly higher sensitivity than HCW-collected nasal specimens. Our results contrast with a prior study finding that throat specimens had a lower sensitivity of 36.8% (compared with 69.4% in our study) for rapid antigen testing.^6^ However, the investigators performed throat swabs only in a small subgroup of 115 of 731 participants included in the study, and a suboptimal sample technique may explain the low sensitivity.^20,21^ Our results also demonstrate that the throat sample technique is more challenging than obtaining a nasal sample, as we found a lower sensitivity and a higher number of inconclusive rapid antigen test results for self-collected throat specimens compared with HCW-collected throat specimens. In contrast, no difference was found between HCW-collected and self-collected nasal specimens. Still, we believe that self-collected throat swabbing can be safely performed, as we did not observe any adverse events (eg, vomiting or choking).

In contrast to the higher SARS-CoV-2 detection rate for both rapid antigen and RT-PCR testing of HCW-collected throat specimens compared with HCW-collected nasal specimens, we found a lower mean Ct value for HCW-collected nasal specimens compared with HCW-collected throat specimens. Our subgroup analysis also revealed that the higher sensitivity of rapid antigen and RT-PCR testing of throat specimens compared with nasal specimens was primarily driven by asymptomatic participants and those with a low viral load (Ct values of 20-34). A study conducted before the Omicron variant also found higher molecular detection of SARS-CoV-2 in throat specimens compared with nasopharyngeal specimens for asymptomatic participants.^5^ Furthermore, an experimental laboratory study^22^ that included healthy volunteer participants injected with a nasal spray of active SARS-CoV-2 still found a higher initial diagnostic sensitivity of throat specimens compared with nasal specimens. These results indicate that the oropharyngeal mucosa may have a higher tissue tropism than previously thought during the COVID-19 pandemic. False-negative nasal test results for individuals with presymptomatic or asymptomatic throat infections may also explain some of the uncontrolled SARS-CoV-2 transmission in the community, as speaking, coughing, and sneezing can generate droplets with the virus.^23^ In contrast, our subgroup analysis indicated that the sensitivity of nasal specimens was comparable to or higher than that of throat specimens for participants who had symptoms for several days before testing. This, combined with the higher viral load, may be explained by SARS-CoV-2 being more massively replicated in the nasal mucosa than in the throat in the later infectious phases.

In agreement with other studies,^2,24,25^ we found comparable sensitivity between self-collected and HCW-collected nasal specimens for rapid antigen testing. However, our study also revealed that adding a throat swab to the current nasal swab recommendations increased the sensitivity of rapid antigen testing of self-collected specimens by 15.5 percentage points. These findings contrast with the FDA advice, which recommends against a throat specimen for rapid antigen testing because the current tests are only authorized with nasal specimens.^3^ Redesigning the current rapid antigen tests to include throat specimens will increase medical manufacturers’ costs and the complexity of home-based rapid antigen testing. However, previous studies support that combined nasal and throat specimens can be self-collected safely with guidance from video instruction,^16,26^ and the National Health Service in the UK has included self-collected throat specimens for rapid antigen testing.^27^ Our findings suggest that the current testing recommendations should include throat specimens to improve the sensitivity of rapid antigen testing. Further research should confirm our findings using redesigned and other rapid antigen testing devices and explore whether throat specimens can also improve the detection of other common airway infections.^28^

### Strengths and Limitations

Major strengths of this study are the randomized design and large sample size, including 2674 participants in the final analysis. The study collected nasal and throat specimens that were analyzed separately by RT-PCR to ensure a highly accurate reference standard for participants with SARS-CoV-2 infection. The rapid antigen tests were then performed with samples from the same anatomical sites to ensure a head-to-head comparison of both the specimen type and the collection method.

Limitations of the study include that the diagnostic performance of home-based testing may be lower because the rapid antigen test result was interpreted by an HCW. Still, we believe our findings can be generalizable to home-based testing because a previous study did not find any difference in the interpretation of rapid antigen testing assays of samples collected by study participants or by HCWs.^24^ Another limitation is that we cannot directly generalize our reported combined nasal and throat specimen sensitivity (estimated by adding the separate rapid antigen test results for the nasal and throat specimens) to a setting where the same swab was used to collect both specimens. However, a previous study supports our conclusions by finding improved sensitivity for rapid antigen testing of specimens from separate nasal and throat swabs as well as using the same swab to collect both specimens.^9^ Lastly, we used 2 different laboratories to perform the RT-PCR analyses, making Ct values from different molecular analyses difficult to compare directly. However, a subgroup analysis of participants with both nasal and throat RT-PCR tests performed in the same laboratory confirmed our overall conclusions (eTable 5 in Supplement 2).

## Conclusions

In this randomized clinical trial of participants from SARS-CoV-2 test centers in Denmark, we found that the sensitivity of SARS-CoV-2 rapid antigen testing was significantly higher for HCW-collected throat specimens than for HCW-collected nasal swabs. For symptomatic individuals, self-collected nasal specimens had a higher sensitivity than self-collected throat specimens. Our results indicate that the throat plays an important role in the initial phase of SARS-CoV-2 infection and that throat specimens should be collected together with nasal specimens to improve sensitivity for rapid antigen testing in both health care and home-based settings.

## Notes

Supplement 1.
Trial Protocol and SAP

Supplement 2. **eAppendix 1.** Questionnaire for Participants **eAppendix 2.** Guide to Self- and HCW-Collected Nasal and Throat Specimens **eAppendix 3.** RT-PCR Method **eAppendix 4.** Sanger Sequencing **eAppendix 5.** Diagnostic Accuracy Calculations **eAppendix 6.** Ethical Approval **eFigure 1.** Flow of Information Through the Different Phases of the Review **eFigure 2.** Specificity of Rapid Antigen Testing **eFigure 3.** Forest Plot of Sensitivity With an Intention-to-Treat Approach **eFigure 4.** Forest Plot With Sensitivity of Rapid Antigen Testing Stratified Based on Symptoms **eFigure 5.** Box Plot With Ct Values Combined With the Correlated Rapid Antigen Results **eTable 1.** Demographics and Clinical Characteristics of the Enrolled and Excluded Participants **eTable 2.** RT-PCR Result for Nasal and Throat Specimens **eTable 3.** SARS-CoV-2 Variants of Concern **eTable 4.** Comparison of Rapid Antigen Testing Results From Different Sites With RT-PCR **eTable 5.** Diagnostic Accuracy Calculated With an Intention-to-Treat Approach of Rapid Diagnostic Antigen Tests **eTable 6.** Subgroup Analysis Comparing RT-PCR and Rapid Diagnostic Antigen Test Results Between the 2 Molecular Laboratories **eTable 7.** Rapid Diagnostic Antigen Test Results Stratified by Symptoms
eReferences

Supplement 3.
Data Sharing Statement
