# Supplementary material for: COVID-19 Rapid Antigen Tests With Self-Collected vs Health Care Worker–Collected Nasal and Throat Swab Specimens: A Randomized Clinical Trial
Source: JAMA Netw Open. 2023 Dec 6;6(12):e2344295. doi: 10.1001/jamanetworkopen.2023.44295 (PMC10701611; doi:10.1001/jamanetworkopen.2023.44295)
Supplement: Supplement 1. — Trial Protocol and SAP [file jamanetwopen-e2344295-s001.pdf]

Diagnostic accuracy of self-performed versus healthcare worker performed anterior nose and oropharyngeal swabs for SARS-CoV-2 detection – study protocol for a randomized controlled trial

Version 2. 19.02 2022

Tobias Todsen[1-3]*, MD, PhD, Kathrine K. Jakobsen[1,3]*, MD, Mathias Peter Grønlund[1], MD, Rasmus E. Callesen[1], MD, Fredrik Folke[3-5], MD, PhD, Helene Larsen[6], PhD, Annette Kjær Ersbøll, PhD[4,7], Thomas Benfield, MD, DMSc[8,3], Tobias Gredal[4], BSN, Mads Klokker[1,3], MD, Nikolai Kirkby[9], PhD, and Christian von Buchwald MD, DMSc[1,3]

* Shared first authorship

1: Department of Otorhinolaryngology, Head and Neck Surgery and Audiology, Copenhagen University Hospital, Rigshospitalet, Copenhagen, Denmark.

2: Copenhagen Academy for Medical Education and Simulation, Capital Region, Copenhagen, Denmark.

3: Department of Clinical Medicine, University of Copenhagen, Denmark.

4: Copenhagen Emergency Medical Services, University of Copenhagen, Copenhagen, Denmark.

5: Department of Cardiology, Copenhagen University Hospital, Herlev Gentofte, Denmark.

6: Center for Diagnostics, Department of Health Technology, Technical University of Denmark (DTU), Denmark

7: National Institute of Public Health, University of Southern Denmark, Copenhagen, Denmark.

8: Department of Infectious Diseases, Copenhagen University Hospital, Amager and Hvidovre, Hvidovre, Denmark.

9: Department of Clinical Microbiology, Rigshospitalet, Copenhagen University Hospital, Copenhagen, Denmark.

Abstract

Background: Extensive testing for COVID-19 during the pandemic is an approach to contain disease spreading. Rapid antigen tests are advantageous by providing quick results and seem manageable for self-performed testing and result interpretation. Thus, the aim of this randomized, controlled trial is to determine the reliability of self-performed sampling for rapid antigen tests compared to sampling performed by healthcare workers. Methods: This study is a non-blinded, two-arm, randomized, controlled trial. The participants are randomized to have specimens collected from the anterior part of the nose and the oropharynx by either oneself or by a healthcare worker for rapid antigen test-ing. In addition, two samples from the same anatomical sites are collected by a healthcare worker and analyzed by RT-PCR. The sensitivity and specificity are calcu-lated and compared across the different test types, and anatomical sites. Results: In expectation, 2934 citizens are required, with a 30% test positive rate, in or-der to detect a test difference of 10%. The sample size will be adjusted if the test positive rate changes. Implication: If self-performed rapid antigen tests turn out to be reliable for COVID-19 testing, this can be a future testing strategy saving time and expenses on personnel and protection equipment while enhancing disease containment.

1. Introduction

Severe acute respiratory syndrome coronavirus 2 (SARS-CoV-2) and the resulting COVID-19 pandemic continue to be a worldwide health emergency [1]–[3] resulting in national lockdown [4]. The golden standard for correct de-tection of SARS-CoV-2 is by reverse transcriptase polymerase chain reaction (RT–PCR) of an upper respiratory specimen. However, this requires laboratory fa-cilities and the analysis is time-consuming and costly. Rapid antigen tests offer a simpler approach, and test results are available within minutes and can thus lead to a faster containment of infection in the society. So far, the specimens for the rapid antigen tests have primarily been collected as nasopharyngeal swabs by trained personnel [5]. However, oropharyngeal sampling for rapid antigen tests is a possible alternative and may be preferred for detection of the Omicron variant [6], [7].

Self-performed testing improves testing access and enables testing outside the healthcare system. Furthermore, self-performed testing reduces the ex-penses for personal protective equipment, and lastly, self-performed testing has been reported to be the preferred sampling method by citizens [8]. Testing with self-collected swab material may however increase the risk of incorrect sampling as the procedure is unsupervised. For this reason, the sensitivity of self-testing compared to testing by professionals has been questioned [9]–[11] and results of the use of self-testing are varying [12], [13]. Studies on rapid antigen tests and self-collected swabs as a screening tool are sparse.
The aim of the study is to compare the diagnostic accuracy between SARS-CoV-2 antigen tests used for self-performed testing to antigen tests per-formed by trained personnel. Furthermore, the diagnostic accuracy of the two sampling methods for antigen tests will be compared with a RT-PCR test on corresponding samples. In addition, we wish to compare the diagnostic accuracy of oropharyngeal-collected specimens versus nasal-collected specimens for both rapid antigen testing and RT-PCR analysis, respectively.

In conclusion, we wish to test the SARS-CoV-2 rapid antigen tests with samples from either the anterior part of the nose or the oropharynx including the palatine tonsils using the same test kits (Standard Q COVID-19 Ag - test, SD Biosensor INC.). The antigen tests will be performed with either self-sampling or samples taken by trained personnel. Furthermore, patients will have two healthcare-worker collected samples from the same anatomical location, the anterior part of the nose and the oropharynx, respectively, for RT-PCR analysis.

Primary research question: In a cohort of individuals from a public test center, what is the diagnostic accuracy of self-collected nasal and throat swabs compared to healthcare worker-collected swabs in the diagnosis of SARS-CoV-2 with antigen tests?
Secondary research question is whether the diagnostic accuracy will be improved by oropharyngeal sampling compared to sampling from the anterior part of the nose in the diagnosis of SARS-CoV-2 using antigen tests and RT-PCR?

2. Materials and Methods

2.1. Study design: We conduct a randomized controlled trial following the CONSORT guidelines.

2.2. Participants: Citizens showing up for a COVID-19 test at Test Center, in the Capital Region are offered to participate in the project on a volunteer basis. In Denmark, citizens are tested for infection with SARS-CoV-2 by swabs of the oropharynx performed by trained personnel, and subsequent analysis of the specimens by a RT-PCR test [7]. The result of the test is given within 24 hours. The oropharyngeal sampling procedure is to swab the palatine tonsils and the posterior wall of the pharynx and subsequently examine it by RT-PCR for SARS-CoV-2 [14].

Inclusion criteria:

Age ≥ 16 years

Exclusion criteria:

Non-fluent in Danish

Impaired citizen i.e. not capable of an independent self-testing

Nasopharyngeal or oropharyngeal anomalies that do not allow for sampling using swabs including neck breathers (tracheostomy/laryngectomy patients)

2.3. Interventions: In addition to the scheduled oropharyngeal swab done by healthcare personnel, sampling from the anterior part of the nose is performed subsequently both intended for RT-PCR analysis using nylon-flocked oropharyngeal and nasopharyngeal swabs (Wuxi NEST Biotechnology Co., Jiangsu, China). Afterward, two rapid antigen tests (Standard Q COVID-19 Ag-test, produced by SD Biosensor INC.) will be performed, one from the anterior part of the nose and one from the oropharynx including the palatine tonsils. Participants are randomized in a 1:1 ratio to whether having specimens for the two rapid antigen tests either self-collected or sampled by trained personnel (Figure 1).

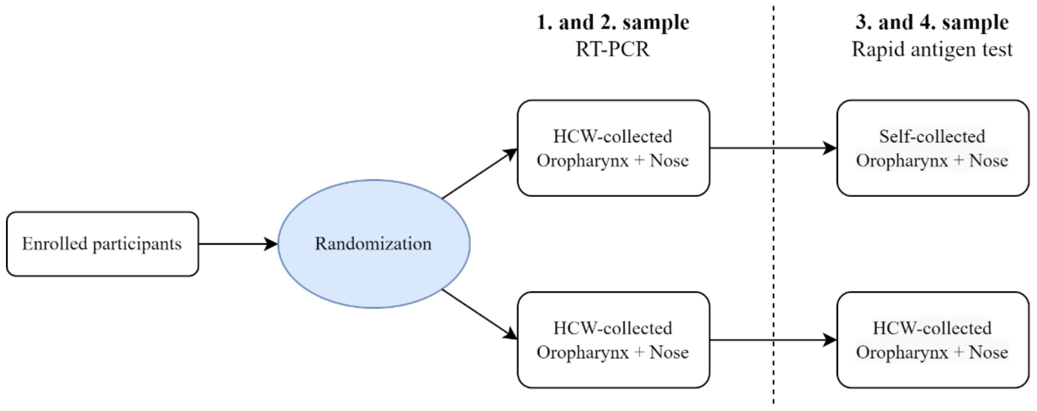

Figure 1. Flowchart of study design.

Abbreviations: RT-PCR: Reverse transcriptase polymerase chain reaction; HCW: Healthcare worker

The swab of the anterior part of the nose is inserted approximately 3-4 cm or until resistance in both nostrils with the same swab according to the manufacturer's instructions [13,14]. The oropharyngeal sampling is performed by collecting specimen from the posterior wall of the oropharynx and both palatine tonsils using the swab from the rapid antigen test kit. This sample is analyzed like the rapid antigen test from the anterior part of the nose. Participants are instructed in the self-testing by means of written instructions and have the opportunity to see a tutorial video accessed by a QR-code.

The participants are registered by the Region's test staff in a secure web database (REDCap) on-site and are questioned regarding information on their symptoms and vaccination status.

All samples intended for RT-PCR analysis are sent to the test facility at the Technical University of Denmark (DTU) or to the Department of Clinical Microbiology, Rigshospitalet for SARS-CoV-2 RT-PCR testing targeting two segments of the Nucleoprotein gene. Analysis results from DTU are electronically transferred to Department of Clinical Microbiology at Rigshospitalet who has personnel responsible for the interpretation and reporting of the result to the participant.

2.4. Clinical outcome: The investigators will define a participant with an RT-PCR positive result as having a COVID-19 infection and this will be the diagnostic reference standard to calculate the accuracy for the self-collected rapid antigen test. The criteria for a positive RT-PCR test result will be a cycle threshold (Ct) value below 34 for at least one of the two gene-targets for SARS-CoV-2. The RNase P ribozyme will be used to assess the presence of human genetic material and considered inconclusive if RNase P Ct > 23 for nasopharyngeal swab (NPS) specimen, and > 27.4 for oropharyngeal swab (OPS) specimen. Participant having an inconclusive RT-PCR test will be excluded from the data analysis.

2.5. Sample size: Based on previous studies the sensitivity of self-testing ranges from 10% to approximately 20% [9]–[11], thus the investigators assume that the sampling performed by trained personnel has a 10% higher sensitivity than self-testing [9]. The prevalence of COVID-19 in Denmark is currently approximately 30% [15]. With this prevalence we expect that inclusion of 2794 participants will provide the study with an 80% power and a 5% significance level. We anticipate that 5% of participants will be lost to follow up (e.g. missing data) thus we require a minimum of 2934 participants, corresponding to including 880 participants with a positive sample. However, if the prevalence varies this will change the required sample size to provide appropriate power for assessing the primary outcome of comparing self-sampling to healthcare collected samples (Table 1).

Table 1: Sample size calculations depending on prevalence, with 80% power and 5% significance level.

Prevalence	10	15	20	25	30	35	40
Participants	10615	6705	4749	3576	2794	2235	1816

Test-positive rates will therefore be monitored during the study period to ensure that our sample size calculation assumptions remain correct.

2.6. Randomization sequence: The randomization list is generated by a computer program (https://www.sealedenvelope.com/simple-randomiser/v1/lists). Participants are randomized at enrollment in block sizes of 40 participants. The table with randomization numbers is operated by and only available to specified personnel at the Department of Otorhinolaryngology, Head and Neck Surgery and Audiology, Rigshospitalet.

2.7. Statistics:
See the Statistical Analysis Plan (SAP) in Appendix

2.8. Ethical considerations:

The primary outcome will be reported as:

Sensitivity and specificity of the self-performed nasal and oropharyngeal swabs for rapid antigen test compared to healthcare worker performed rapid antigen test

Secondary outcome:

The sensitivity of the RT-PCR tests of nasal and throat specimens.

SARS-CoV-2 RT-PCR cycle threshold (Ct) value of nasal and throat specimens

The rapid antigen test sensitivity for participants with symptoms and low RT-PCR cycle threshold (Ct) values

2.9. Ethical considerations: There are no known risks associated with participation in the project. The protocol complies with the Declaration of Helsinki II. The protocol was reported to the Regional Ethics Committee of the Capital Region of Denmark (P-2022-47) and was considered exempt from further processing (protocol no. H-21059629 and 21074917).

2.10. Recruitment of participants and informed consent: All patients aged 16 years or older showing up for a COVID-19 test at RegionH´s test facility in Copenhagen Airport are offered to participate in the project. Upon arrival, the participants will receive oral and written information about the project, as well as written information about subjects' rights. The right to bring a counsel to the information interview will likewise be explained. The information interview is carried out by the staff from the Capital Region. The participants are informed that they can withdraw their consent at any time without the affection of further processes or potential treatment. Only citizens who volunteer to participate and sign the informed consent form will be enrolled. Participants will be offered no financial compensation for their participation.

2.11. Side effects, risks, and disadvantages for patients: In total, sampling is performed four times from each patient; twice from the nose and twice from the oropharynx. Besides the potential discomfort associated with the sampling procedure itself, there will be a minor inconvenience for the patients in terms of the time spent on the examination [16].

2.12. Clinical information from patient records: Age of the participants, the reason for testing for COVID-19, as well as the results from the PCR test and the rapid antigen test will be registered for all trial participants.

2.13. Funding: This is an investigator-initiated clinical trial. No members of the research group behind the project have financial interests in the execution or results of the project. The test staff who are to carry out the swabs for RT-PCT testing are part of Test center Danmark's staff and are paid from here. A grant from the Novo Nordisk Foundation (Grant number: NNF21SA0069151) covered the salary for the research staff involved in the project. The rapid antigen tests and the expenses of the RT-PCR analyses are made available without payment by the distributor (Copenhagen Medical A/S, Copenhagen, Denmark), however they have no role in the study design, data interpretation or writing of the manuscript.

3. Perspectives

If the sensitivity of the self-performed rapid antigen tests turns out to be reliable, this may lead to a more widespread use of the rapid antigen tests in detection of COVID-19 infection as well as other upper respiratory tract infec-tions. As the response time is significantly faster for rapid antigen tests than for PCR testing, it can potentially lead to a quicker containment of infection, and thus a better opportunity to bring COVID-19 infection under control. In addition, home-testing for COVID-19 with rapid antigen tests can increase testing fre-quency and number of people being tested as well as save the time and expenses of health care personnel performing the sampling.

As the COVID-19 pandemic develop to an epidemic, the self-performed rapid antigen tests could prove useful for quick implementation of citizen screening as well as testing for COVID-19 infection prior to contact with the health care system. Also, rapid antigen tests could be useful for general prac-titioners to contain infection in a subpopulation rapidly.

A secondary outcome of the study is to examine the usefulness of oro-pharyngeal sampling for rapid antigen tests. This, in order to find the most sensitive sampling method for future home-testing.

References

[1] "Analyser og prognoser for epidemiens udvikling." https://covid19.ssi.dk/analyser-og-prognoser/analyser-og-prognoser-for-epidemien-udvikling (accessed Dec. 16, 2020).

[2] "Ugentlige opgørelser med overvågningsdata." https://covid19.ssi.dk/overvagningsdata/ugentlige-opgorelser-med-overvaagningsdata (accessed Dec. 16, 2020).

[3] "Weekly operational update on COVID-19 - 21 December 2020." https://www.who.int/publications/m/item/weekly-operational-update-on-covid-19---21-december-2020 (accessed Dec. 22, 2020).

[4] SSI, "Covid-19: testing strategy," 2020. %0Atcdk.ssi.dk

[5] K. K. Jakobsen *et al.*, "Accuracy and cost description of rapid antigen test compared with reverse transcriptase-polymerase chain reaction for sars-cov-2 detection," *Danish Medical Journal*, vol. 68, no. 7, Jul. 2021.

[6] D. A. Olsen *et al.*, "Quantifying SARS-CoV-2 nucleocapsid antigen in oropharyngeal swabs using single molecule array technology," *Scientific Reports*, vol. 11, no. 1, p. 20323, Dec. 2021, doi: 10.1038/s41598-021-99807-7.

[7] G. Marais *et al.*, "Saliva swabs are the preferred sample for Omicron 1 detection 2 3", doi: 10.1101/2021.12.22.21268246.

[8] J. S. Bundgaard *et al.*, "Danish citizens' preferences for at-home oropharyngeal/nasal SARS-CoV-2 specimen collection," *International Journal of Infectious Diseases*, vol. 109, pp. 195–198, Aug. 2021, doi: 10.1016/j.ijid.2021.06.060.

[9] D. J. McCulloch *et al.*, "Comparison of Unsupervised Home Self-collected Midnasal Swabs With Clinician-Collected Nasopharyngeal Swabs for Detection of SARS-CoV-2 Infection," *JAMA network open*, vol. 3, no. 7, p. e2016382, Jul. 2020, doi: 10.1001/jamanetworkopen.2020.16382.

[10] S. Würstle *et al.*, "Self-sampling versus health care professional-guided swab collection for SARS-CoV-2 testing," *Infection*, vol. 49, no. 5, pp. 927–934, Oct. 2021, doi: 10.1007/s15010-021-01614-9.

[11] A. Harmon *et al.*, "Validation of an At-Home Direct Antigen Rapid Test for COVID-19," *JAMA Network Open*, vol. 4, no. 8, Aug. 2021, doi: 10.1001/jamanetworkopen.2021.26931.

[12] A. K. Lindner *et al.*, "Head-to-head comparison of SARS-CoV-2 antigen-detecting rapid test with professional-collected anterior nasal versus nasopharyngeal swab," *medRxiv*, pp. 2–9, 2020, doi: 10.1101/2020.12.03.20243725.

[13] S. Tonen-Wolyec, R. Dupont, N. Awaida, S. Batina-Agasa, M. P. Hayette, and L. Bélec, "Evaluation of the practicability of biosynex antigen self-test covid-19 ag+ for the detection of sars-cov-2 nucleocapsid protein from self-collected nasal mid-turbinate secretions in the general public in france," *Diagnostics*, vol. 11, no. 12, Dec. 2021, doi: 10.3390/diagnostics11122217.

[14] "Hurtigtest vejledninger - Region Midtjylland." https://www.rm.dk/om-os/indkob--medicoteknik/hurtigtest-vejledninger/ (accessed Jan. 11, 2022).

[15] S. og S. S. Institut, “Coronatal: nøgletal, indlæggelser og kapacitet - Sundhedsstyrelsen.” https://www.sst.dk/da/corona/status-for-epidemien/tal-og-overvaagning (accessed Feb. 19, 2022).

[16] J. H. Therchilsen *et al.*, “Self-Collected versus Healthcare Worker-Collected Swabs in the Diagnosis of Severe Acute Respiratory Syndrome Coronavirus 2,” *Diagnostics*, vol. 10, no. 9, pp. 1–10, 2020, doi: 10.3390/diagnostics10090678.

Statistical Analysis Plan

Statistical Analysis Plan (SAP)

Title: Diagnostic accuracy of SARS-CoV-2 rapid antigen tests with self-collected vs healthcare-collected nasal and throat swabs – a multicenter, randomized clinical trial.

Senior statistician: Professor Annette Kjær Ersbøll, PhD
Chief investigators: Associate Professor Tobias Todsen, MD, PhD and Kathrine Kronberg Jakobsen, MD

This SAP follows the reporting recommendation from “Guidelines for the Content of Statistical Analysis Plans in Clinical Trials.” by Gamble C, Krishan A, Stocken D, Lewis S, Juszczak E, Doré C, et al. published in JAMA 2017;318:2337-43.

Introduction

1.1 Background and rationale

Rapid antigen testing is critical to identify cases of coronavirus disease 2019 (COVID-19). It remains unclear whether a self- or healthcare worker (HCW) collected nasal, throat swab or a combination is the most sensitive sampling method to detect severe acute respiratory syndrome coronavirus 2 (SARS-CoV-2).

1.2 Objectives

The main objective is to compare the diagnostic accuracy of rapid antigen testing for SARS-CoV-2 using self- vs healthcare worker (HCW) collected nasal and throat swabs.

Study Methods

2.1 Trial design

A randomized, controlled multicenter trial will be conducted with participants being randomized to either self or HCW nasal and throat swabbing for rapid antigen testing. Reverse transcriptase polymerase chain reaction (RT–PCR) testing of HCW-collected nasal and throat swabs will be used as the reference standard to calculate the sensitivity and specificity of rapid antigen testing. A study participant will be considered positive if SARS-CoV-2 was detected in at least one upper respiratory specimen by RT-PCR (gold standard).

2.2 Randomization

Enrolled participants were randomized in a 1:1 ratio either to self-collect, or to have HCW-collected nasal and throat specimens for RDT. Block randomization was performed prior to the enrollment using an online randomization program[1] and the type of intervention was disclosed in connection with trial registration in Redcap.

Sample size

We expect to find a 10% difference in the rate of SARS-CoV-2 detection for self vs HCW-collected swabs (13) and a prevalence of SARS-CoV-2 infection of 30%. With a beta of 0.8 and alpha of 0.05 we plan to enroll a total of 2,794 participants.

Framework

Our hypothesis is that HCW swabbing is superior to self-collected nasal and throat swabs.

2.5 Statistical interim analysis and stopping guidance

All the statistical analyses will be conducted after the number of participants enrolled reaches 2,794 participants and no statistical interim analysis will be conducted.

2.6 Timing of final analysis

All outcomes from the trial were analyzed at the same time after the study ended.

Statistical principles

3.1 Confidence intervals and *P* values

The level of statistical significance will be $p < 0.05$ and 95% confidence interval will be reported.

3.2 Adherence and Protocol deviations

Definition of adherence to the intervention

Adherence is defined as participants who has a full registration of identification number (CPR number), test center site for collection of specimen and randomization to control or intervention group. Further, the participants need to have valid test results from all the nasal and throat RT-PCR and rapid antigen tests to be included in the final data analysis. Compliance is assessed based on the number and percentage of subjects who have correct registration information and valid test results.

Description of adherence

The adherence to the intervention will be summarized in the study flowchart.

Definition of protocol deviations for the trial

The participants will be excluded from final analysis if one or more of the following deviations from the testing protocol was found:

Missing identification number (CPR number), no registered test center sites or no randomization (intervention or control) registered.

Age below 17

Participants included more than once only contribute with samples from the first test date

Missing or invalid rapid antigen tests results

Missing RT-PCR tests results

Description of which protocol deviations will be summarized

The number and type of protocol deviation will be registered, and the number of participants removed will be summarized in the CONSORT flow diagram.

3.3 Analysis populations
A complete case analysis strategy will be used to select the participants included in the final statistical analysis.

Trial Population

4.1 Screening data
We aim to invite individuals from Kastrup and Valby Covid-19 test centers to represent citizens from two urban areas in Copenhagen, Denmark to participate in the study.

4.2 Eligibility
All individuals being 18 years or older will be invited to participate in the trial. The same individual will only be allowed to participate in the study once.

The exclusion criteria were individuals with a tracheostomy, laryngectomy, or prior oropharyngeal cancer surgery and individuals without a Danish civil registration number (CPR).

4.3 Recruitment
A CONSORT flow diagram will be used to summarize the number of included participants with information about:
Total number of Covid-19 tested individuals during the study period
Number of participants lost to identify / registrar
Number of participants excluded from final analyses due missing test results
4.4 Withdrawal/Follow-up

The level of withdrawal and the missing final RT-PCR and rapid antigen test results during the study will be tabulated.

4.5 Baseline patient characteristics
List of baseline characteristics for participants:

Measure	Outcome	Description
Demographic data	Age and gender	Data from the Danish Civil Registration System
Test center	Kastrup or Valby Covid-19 test center	Registration of the test center where the participants were enrolled
Questionnaire	Test reason, symptom description and length, vaccination status	Questionnaire registered in RedCap

Categorical data will be summarized with number and percentage while continuous data will be summarized by mean and standard deviation or median and interquartile range (Q_1, Q_3). We will not perform tests of statistical significance for baseline characteristics.

Analysis

5.1 Outcome definitions
The primary outcome:
The sensitivity and specificity of rapid antigen tests of self- and HCW-collected nasal and throat specimens.

The secondary outcome:
The sensitivity of the RT-PCR tests of nasal and throat specimens.
SARS-CoV-2 RT-PCR cycle threshold (Ct) value of nasal and throat specimens.

5.2 Analysis methods
Analysis method and treatment effects
Differences in the proportion of SARS-CoV-2 positive rapid antigen test (among participations with a positive RT-PCR test) between the collection methods (self- vs HCW-collected) will be compared using binary logistic regression including test center as a fixed effect stratified by specimen. Differences in the proportion of SARS-CoV-2 positive rapid antigen test between the specimen types will be compared using binary logistic regression and a generalized estimating equation to adjust for clustering of observations within participant (nasal and throat swabs). Test center will be included as a fixed effect. The Ct values from positive RT–PCR samples will be compared using a general

linear mixed model with a random effect of participant. Ct values will be logarithmic transformed to account for a skewed distribution if necessary.
The 95% confidence intervals (CI) will be presented. The level of statistical significance will be defined as $p < 0.05$.

Adjustment for covariates
The regression analyses will be adjusted for the effect of the test centers.

Methods used for assumptions to be checked for statistical methods
Assumptions for the logistic regression analysis included a binary outcome, independent observations, and linearity in logit for continuous variables. To account for lack of independent observations, a generalized estimating equation approach and mixed models will be applied. No continuous variables will be included.

Assumptions for the linear regression analysis included a normal distribution, independent observations, equal variation (homoscedasticity) and linearity for continuous variables. Normally distributed outcomes and homoscedasticity will be evaluated visually by plots of the residuals. To account for lack of independent observations, a generalized estimating equation approach and mixed effect models will be applied. No continuous variables will be included.

Details of alternative methods to be used if distributional assumptions do not hold, e.g., normality, proportional hazards, etc.
If the assumption of a normal distribution of the outcome in the linear regression model is not fulfilled, a transformation of the outcome will be applied (e.g., logarithmic or rank transformations).

Planned sensitivity analyses for each outcome
We planned to do sensitivity analyses using two N-gene segments (with cycle threshold (Ct) < 30) to define the true positive SARS-CoV-2 infections in our study. This was done to explore the consequences of using a higher test specificity on the overall diagnostic results compared to the per protocol definition of positive for SARS-CoV-2 infection using cycle threshold (Ct) < 34 for at least one N-gene segment. Further, we also planned to test the robustness of our findings by performing the statistical analysis with definition of the inconclusive rapid antigen tests as negative to explore a potential bias from the distribution of the inconclusive result. Further we did also perform a Bayesian latent class analysis for accounting for an imperfect reference standard and estimate the sensitivity and specificity for RT-PCR of nasal and throat specimens.

Planned subgroup analyses
Further, we planned to do subgroup analyses exploring the distribution of positive test results for participants stratified by symptoms and molecular laboratory performing the RT-PCR tests.

5.3 Missing data

Participants who will not adhere to the intervention definition (see SAP) will be reported as missing data and excluded from final analysis. Participants with missing data for the baseline characteristics from the questionnaire will be included in the statistical analysis of primary outcome and secondary outcomes. A table with baseline characteristics will be presented as raw data.

5.4 Additional analyses

Not applicable.

5.5 Harms

Any adverse events during or after the collection of respiratory specimens for the trial will be noted and categorized into acute bleeding or foreign body in upper airway.

5.6 Statistical software

SAS statistical software suite ver. 9.4 (SAS Institute, North Carolina, U.S.) and R.
