# Supplementary material for: COVID-19 Rapid Antigen Tests With Self-Collected vs Health Care Worker–Collected Nasal and Throat Swab Specimens: A Randomized Clinical Trial
Source: JAMA Netw Open. 2023 Dec 6;6(12):e2344295. doi: 10.1001/jamanetworkopen.2023.44295 (PMC10701611; doi:10.1001/jamanetworkopen.2023.44295)
Supplement: Supplement 2. — eAppendix 1. Questionnaire for Participants eAppendix 2. Guide to Self- and HCW-Collected Nasal and Throat Specimens eAppendix 3. RT-PCR Method eAppendix 4. Sanger Sequencing eAppendix 5. Diagnostic Accuracy Calculations eAppendix 6. Ethical Approval eFigure 1. Flow of Information Through the Different Phases of the Review eFigure 2. Specificity of Rapid Antigen Testing eFigure 3. Forest Plot of Sensitivity With an Intention-to-Treat Approach eFigure 4. Forest Plot With Sensitivity of Rapid Antigen Testing Stratified Based on Symptoms eFigure 5. Box Plot With Ct Values Combined With the Correlated Rapid Antigen Results eTable 1. Demographics and Clinical Characteristics of the Enrolled and Excluded Participants eTable 2. RT-PCR Result for Nasal and Throat Specimens eTable 3. SARS-CoV-2 Variants of Concern eTable 4. Comparison of Rapid Antigen Testing Results From Different Sites With RT-PCR eTable 5. Diagnostic Accuracy Calculated With an Intention-to-Treat Approach of Rapid Diagnostic Antigen Tests eTable 6. Subgroup Analysis Comparing RT-PCR and Rapid Diagnostic Antigen Test Results Between the 2 Molecular Laboratories eTable 7. Rapid Diagnostic Antigen Test Results Stratified by Symptoms eReferences [file jamanetwopen-e2344295-s002.pdf]

Supplementary Online Content

Todsen T, Jakobsen KK, Grønlund MP, et al. COVID-19 rapid antigen tests with self-collected vs health care worker–collected nasal and throat swab specimens: a randomized clinical trial. *JAMA Netw Open*. 2023;6(11):e2344295. doi:10.1001/jamanetworkopen.2023.44295

eAppendix 1. Questionnaire for Participants
eAppendix 2. Guide to Self- and HCW-Collected Nasal and Throat Specimens
eAppendix 3. RT-PCR Method
eAppendix 4. Sanger Sequencing
eAppendix 5. Diagnostic Accuracy Calculations
eAppendix 6. Ethical Approval
eFigure 1. Flow of Information Through the Different Phases of the Review
eFigure 2. Specificity of Rapid Antigen Testing
eFigure 3. Forest Plot of Sensitivity With an Intention-to-Treat Approach
eFigure 4. Forest Plot With Sensitivity of Rapid Antigen Testing Stratified Based on Symptoms
eFigure 5. Box Plot With Ct Values Combined With the Correlated Rapid Antigen Results
eTable 1. Demographics and Clinical Characteristics of the Enrolled and Excluded Participants
eTable 2. RT-PCR Result for Nasal and Throat Specimens
eTable 3. SARS-CoV-2 Variants of Concern
eTable 4. Comparison of Rapid Antigen Testing Results From Different Sites With RT-PCR
eTable 5. Diagnostic Accuracy Calculated With an Intention-to-Treat Approach of Rapid Diagnostic Antigen Tests
eTable 6. Subgroup Analysis Comparing RT-PCR and Rapid Diagnostic Antigen Test Results Between the 2 Molecular Laboratories
eTable 7. Rapid Diagnostic Antigen Test Results Stratified by Symptoms
eReferences

This supplementary material has been provided by the authors to give readers additional information about their work.

eAppendix 1 Questionnaire for participants

General data	
CPR number	______________ (DDMMYY-XXXX)
Today´s date	______________
Questionnaire regarding COVID-19	
Have you been vaccinated against COVID-19?	○ Yes<br>○ No
Why have you booked an appointment for a COVID-19 test?	○ I have COVID-19-like symptoms (e.g., fever, general tenderness, sore throat, cough, fatigue, diarrhea, headache, loss of taste or smell, skin rash, eye cataracts, shortness of breath)<br>○ I have been in contact with an infected person<br>○ I follow the recommendations of regular testing.<br>○ Other
How long have you had symptoms?	○ One day<br>○ Two days<br>○ Three days<br>○ Four days<br>○ Five days<br>○ Six days or more
Comment	______________

© 2023 Todsen T et al. *JAMA Network Open*.

GUIDE TIL SELF-COLLECTED THROAT SWAB

1. Start disinfecting your hands and swallowing any food or liquid in your mouth before the swab procedure.
2. Unpack the swab from the packaging and hold the swab firmly. Use your phone to light up in your mouth before the next step.
3. Stick out your tongue and use the mirror for visual display. Locate your tonsils(*) and the back wall of the pharynx (*).
4. The swab is inserted directly on the back wall of the throat, where the swab should be gently pressed and rotated. The swab should also include one of the tonsils before being removed.
5. The swab should avoid touching the tongue or cheeks during the procedure.
6. After the self-sampling, insert the swab into the extraction buffer tube. Squeeze the buffer tube while the swab is stirred to release the virus into the buffer. The swab is then pulled up and discarded.
7. Press the nozzle cap onto the tube, apply four drops of the extracted specimen to the well of the rapid antigen test device, and call for an employee.

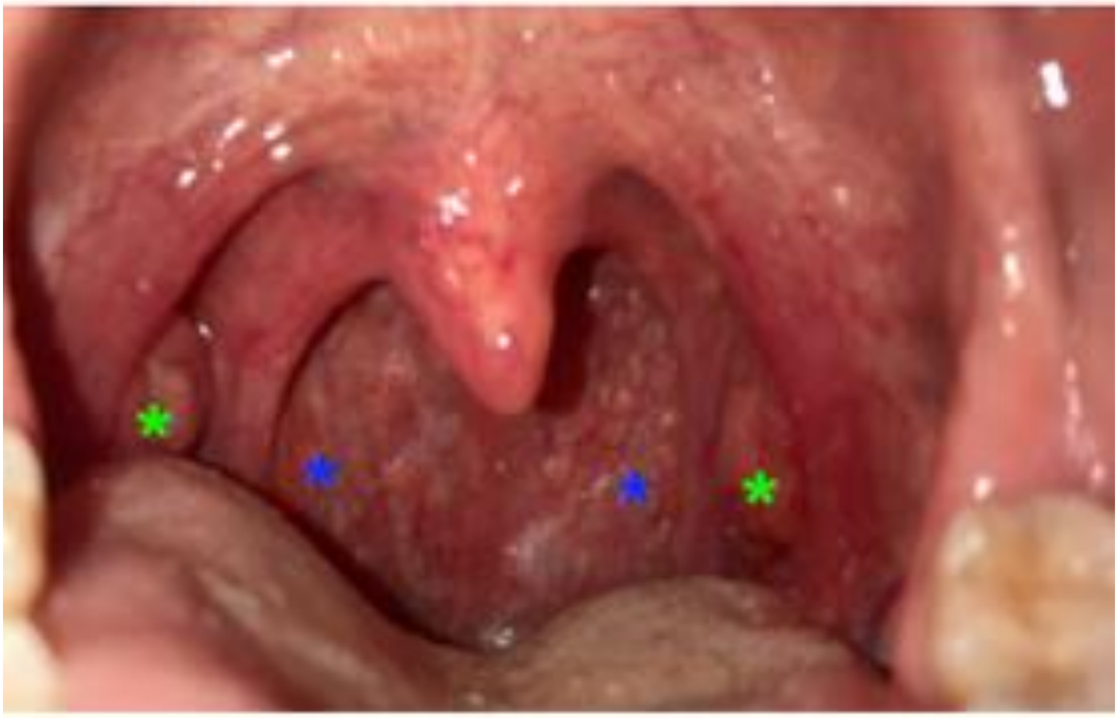

See www.urt-sample.com for pictures and online educational videos

GUIDE TIL SELF-COLLECTED NASAL SWAB

© 2023 Todsen T et al. *JAMA Network Open*.

1. Blow your nose with a tissue and disinfect your hands.
2. Unpack the swab from the packaging and hold the swab firmly.
3. Insert the swab minimum of 2 cm into any nostril, turn it three times, and take it out. The swab should be inserted horizontally along the floor of the nose and not upwards.
4. The procedure is then repeated in the other nostril.
5. After the self-sampling, insert the swab into the extraction buffer tube. Squeeze the buffer tube while the swab is stirred to release the virus into the buffer. The swab is then pulled up and discarded.
6. Press the nozzle cap onto the tube, apply four drops of the extracted specimen to the well of the rapid antigen test device, and call for an employee.

See www.urt-sample.com for pictures and online educational videos

GUIDE TO HCW-COLLECTED THROAT SWAB

1. Ensure you are on the same level as the patient to have a good view of the oropharynx. Ask the patient to take off the mask during the procedure.
2. Hold the swab like a pencil between the thumb and the first and second fingers.
3. Ask the patient to open the mouth and say “aaah” so the soft palate will rise.
4. Insert the swab and swipe with rotating movements over one tonsil (1), the posterior wall of the oropharynx (2), and finish by swabbing the other tonsil (3).
5. Withdraw the swab without touching the cheeks, the teeth, or the gums. If the tongue obstructs the visualization of the posterior wall of the oropharynx, use a tongue depressor to get a better view.
6. After the sampling, insert the swab into the extraction buffer tube. Squeeze the buffer tube while the swab is stirred to release the virus into the buffer. The swab is then pulled up and discarded.
7. Press the nozzle cap onto the tube and apply four drops of the extracted specimen to the well of the rapid antigen test device.

© 2023 Todsen T et al. *JAMA Network Open*.

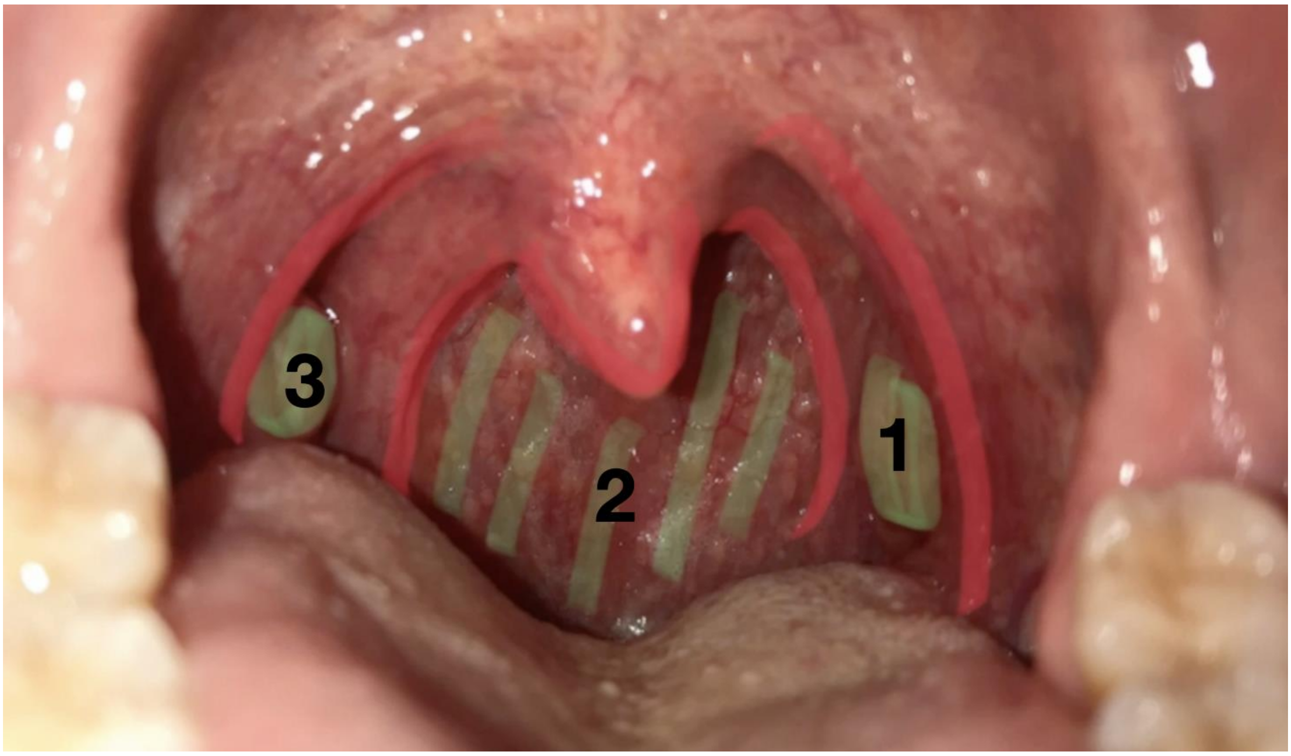

See www.urt-sample.com for pictures and online educational videos

GUIDE TO HCW-COLLECTED NASAL SWAB

1. Ask the patient to tile the head slightly back.
2. Insert the swab 2-4cm into the nostril (or until resistance is met) in a direction toward the ear lobe (avoid pointing the swab upwards as it can be unpleasant for the patient).
3. Rotate the swab three times against the nasal wall.
4. Take out the swab while rotating and repeat the procedure on the opposite nostril
5. After the sampling, insert the swab into the extraction buffer tube. Squeeze the buffer tube while the swab is stirred to release the virus into the buffer. The swab is then pulled up and discarded.
6. Press the nozzle cap onto the tube and apply four drops of the extracted specimen to the well of the rapid antigen test device.

See www.urt-sample.com for pictures and online educational videos

© 2023 Todsen T et al. *JAMA Network Open*.

eAppendix 3 RT-PCR method

The throat and nose swabs for RT-qPCR were collected with a sterile Oropharyngeal Collection Swab (Wuxi NEST Biotechnology Co., Ltd, Wuxi City, China) with 22 mm swab head of flocked nylon and 150 mm shaft of ABS. The swab heads with specimens were stored in separate sterile tubes with 2mL of inactivation transport medium (Wuxi NEST Biotechnology Co., Ltd, Wuxi City, China). The samples were sent for RT-qPCR testing at the Technical University of Denmark, Lyngby, Denmark (DTU) or to the Department of Clinical Microbiology, Rigshospitalet for SARS-CoV-2 RT-PCR testing targeting two segments of the nucleocapsid (N) gene. At DTU, the RNA was purified from samples using RNAdvance Viral extraction kit (cat. No. C57956, Ramcon) and samples were PCR analyzed with the CoviDetect - COVID-19 multiplex RT-qPCR assay from PentaBase (Odense, Denmark).

Oligo	Sequence (5'→ 3')
2019-NCOV_N1-FW	GACCCCAAAATCAGCGAAAT
N1.RV4	CGCAGTATTATTGGGTAAACC
N1.P.HE1	FAM-TGCACCCCGCATTACGTTTG-BHQ1
NCOV N2.FW2	AGGAACTGATTACAAACATTGGC
N2.RV2	TGTAGGTCAACCACGTTCCC
N2.HE.PROBE4.PY	PY-TGCACAATTTGCCCCCAGG-BHQ1
RNP.FW3	CGG TGT TTG CAG ATT TGG AC
RNP.REV2DSUP	GGC TGT CTC CAC AAG TC
RPN.HE_PROBE1	CY5-CGGGTTCTGACCTGAAGG-BHQ2

The primer sequences are shown without modifications designed by Pentabase as these are non-disclosure material.

CoviDetect targets N1/N2 nucleocapsid viral genes and a human target RNAse P (for monitoring proper sampling).

RT-PCR program, CovidDetect:

No.	Step	Temperatur	Acquisition	Time	Cycles
1	Reverse transcription	52 °C	-	5 min.	1
2	Initial PCR activation	95 °C	-	10 sec.	1
3	Denaturation	95 °C	-	5 sec	7
4	Anneling	66 °C	-	30 sec	

© 2023 Todsen T et al. *JAMA Network Open*.

6	Denaturation	95 °C	-	5 sec	38
7	Anneling	60 °C	GREEN (N1), YELLOW (N2), RED (RP)	30 sec	

The fluorescence level is not recorded during the first 7 cycles of the RT-qPCR protocol from PentaBase, which results in Ct values that are approximately 7 Ct lower compared with regular COVID RT-qPCR protocols. Thus the Ct results can be adjusted by adding 7 Ct to the result. Results were automatically uploaded to Rigshospitalets database and inconclusive samples (negative for all targets) were validated by a laboratory scientist at DTU and sent for retest. A molecular biologist or a clinical microbiologist at Rigshospitalet validated result registration. An RT-PCR test was deemed positive if cycling threshold (Ct) was 34 or below for at least one of two gene targets of SARS-CoV-2 (gold standard). (1)

© 2023 Todsen T et al. *JAMA Network Open*.

eAppendix 4 Sanger sequencing

The detailed SpikeSeq protocol is available from Jørgensen et al. (2) and also described in detail below.

The RT-qPCR prior to Sanger sequencing was set up in 20 µL reactions using 10 µL AmpliSmaRT One-Step RT-qPCR 2 × Master Mix (PentaBase A/S, Odense, Denmark), 5 µL 4 × primer mix and 5 µL eluate from the BasePurifier. The RT-qPCR reaction was performed using the following program: reverse transcription for 5 min at 52 °C, then hot start polymerase activation at 95 °C for 10 s, followed by 45 cycles of 95 °C for 5 s, 58 °C for 30 s, and 72 °C for 1 min, followed by 5 min at 72 °C. 1.5 µL of the unpurified PCR product along with 2 µL 10 µM sequencing primer diluted in 15 µL nuclease free water was shipped to Eurofins Genomics (Eurofins, Cologne, Germany) for Sanger sequencing using their Plate Seq Kit Mix. Two sequencing primers were designed to amplify from amino acid Asp17 and Thr385 in the S gene, covering the lower S protein and the RBD respectively. The sequencing primer described by Jørgensen et al. was used. (2) Approximately 9% of the positive samples had high Ct values (>Ct 28) and did not return any sequence information despite re-analysis. A proportion of samples with Ct values < Ct 28 did not return any sequence information, despite re-analysis, and we predict that this is due to poor RNA integrity.

eAppendix 5 Diagnostic accuracy calculations

Terminology	Definition
SARS-CoV-2 true positive (P)	The number of participants with positive RT-PCR test from either nasal, throat, or both specimens
SARS-CoV-2 true negative (N)	The number of participants with negative RT-PCR test results for both nasal and throat swabs
True positive (TP)	A positive rapid antigen test result on a SARS-CoV-2 true positive participant (P)
True negative (TN)	A negative rapid antigen test result on a SARS-CoV-2 true negative participant (N)
False positive (FP)	A positive rapid antigen test result on a SARS-CoV-2 true negative participant (N)
False negative (FN)	A negative rapid antigen test on a SARS-CoV-2 true positive participant (P)

Sensitivity = TP / (TP + FN)

Specificity = TN / (TN + FP)

Positive predictive value (PPV) = TP / (TP + FP)

Negative predictive value (NPV) = TN / (TN + FP)

© 2023 Todsen T et al. *JAMA Network Open*.

eAppendix 6 Ethical approval

Region Hovedstaden

Center for Regional Udvikling

De Videnskabsetiske Komiteer

Blegdamsvej 60, 1. sal, opgang 94A11
2100 København Ø

Afsnit Sundhedsforskning og Innovation
Telefon 3866 6395
Mail vek@regionh.dk

Journal-nr.: 22012313

Dato: 21-02-2022

Tobias Todsen
Rigshospitalet
Øre-næse-halskirurgisk og Audiologisk Klinik
Blegdamsvej 9
2100 København Ø

22012313 - Forespørgsel om anmeldelsespligt til videnskabetisk komite, tilføjelse af podning fra munden

Projekt: Undersøgelse af sensitiviteten ved selvpodning med COVID-19 antigen quick test af forreste del af næsen i Dansk screeningssammenhæng.

Du har ved mail af 25. januar 2022 spurgt, om ovennævnte projekt skal anmeldes til det videnskabsetiske komitesystem.

Projektet er den 16. december 2021 vurderet ikke anmeldelsespligtigt (journalnummer 21074917), og der ønskes nu tilføjelse af podning fra munden.

Det er oplyst, at borgere, der får en COVID-19 PCR-test, spørges om at udføre en selvtest med podning i næsen og munden som kviktest samt podning af forreste del af næsen af sundhedsfaglig person til PCR-undersøgelse. Resultatet af ekstra test valideres ved den standard PCR-test, der tages under alle omstændigheder.

Det er vurderet, at den ekstra podning fra næse og mund er en sundhedsvidenskabelig intervention af så lille en størrelsesorden, at der ikke er tale om et sundhedsvidenskabeligt forskningsprojekt, som dette er defineret i komitélovens § 2, og at selve analysen er metodevalidering, da testresultatet udelukkende skal holdes op imod den PCR-test, der foretages uanset om borgeren deltager i forsøget eller ej.

Projektet er derfor fortsat ikke anmeldelsespligtigt, jf. komitélovens § 1, stk. 4 og kan iværksættes uden tilladelse fra De Videnskabsetiske Komiteer for Region Hovedstaden.

I Danmark har det videnskabsetiske komitesystem til opgave at vurdere sundhedsvidenskabelige og sundhedsdatavidenskabelige forskningsprojekter.

Ved sundhedsvidenskabelige forskningsprojekter forstås projekter, der indebærer forsøg på levendefødte menneskelige individer, menneskelige kønsceller, der agtes anvendt til befrugtning, menneskelige befrugtede æg, fosteranlæg og fostre, væv, celler og arvebestanddele fra mennesker, fostre og lign. eller afdøde. Herunder omfattes kliniske forsøg med lægemidler på mennesker og klinisk afprøvning af medicinsk udstyr.

© 2023 Todsen T et al. *JAMA Network Open*.

Sundhedsvidenskabelig forskning omhandler primært forskning inden for de lægevidenskabelige fag, den kliniske og den socialmedicinsk-epidemiologiske forskning. Begrebet omfatter, udover forskning af de somatiske sygdomme, tillige de psykiatriske og de klinisk-psykologiske sygdomme og tilstandsformer. Herudover inddrages tilsvarende odontologisk og farmaceutisk forskning under begrebet.

Registerforskningsprojekter (bortset fra sundhedsdatavidenskabelige projekter), interviewundersøgelser og spørgeskemaundersøgelser skal kun anmeldes, hvis der indgår menneskeligt biologisk materiale i projektet.

Undersøgelser af anonymt biologisk humant materiale skal dog ikke anmeldes til en videnskabsetisk komite, med mindre der er tale om et forskningsprojekt vedrørende befrugtede menneskelige æg samt kønsceller, jf. §§ 25 og 27, stk. 2 i lov om kunstig befrugtning i forbindelse med lægelig behandling, diagnostik og forskning m.v. Det er et krav, at materiale er fuldstændig anonymt (der må ikke være en identifikationskode til data), og at materialet er indsamlet i overensstemmelse med lovgivningen på indsamlingsstedet.
Forsøg på cellelinjer eller lignende, der stammer fra et forsøg med indsamling af celler eller væv, som har opnået den nødvendige godkendelse, skal heller ikke anmeldes.

Forsøg, der alene har til formål at fastlægge et kemikaliums toksikologiske grænse i mennesket, er ikke anmeldelsespligtige. Ved et kemikalium forstås i denne forbindelse et stof, der ikke finder terapeutisk anvendelse.

Der ligger således ikke i afvisningen af at bedømme projektet nogen etisk stillingtagen eller negativ vurdering af dets indhold.

Ved sundhedsdatavidenskabelige forskningsprojekter forstås forskning vedrørende særlige komplekse områder i afledte sensitive bioinformatiske data frembragt ved omfattende kortlægning af arvemassen eller billeddiagnostik i forbindelse med forsøg eller klinisk diagnostik af patienter.

Vi gør opmærksom på, at regionerne i visse tilfælde skal godkende videregivelse af oplysninger fra patientjournaler. Det er den region, forsker er ansat i, der skal ansøges om dette. Nærmere oplysninger kan findes på den relevante regions hjemmeside.

Behandling af personhenførbare oplysninger er omfattet af databeskyttelsesloven/persondataforordningen. Nærmere oplysning herom findes på Datatilsynets hjemmeside.

Klagevejledning:

Afgørelsen kan, jf. komitélovens § 26, stk. 1, indbringes for National Videnskabsetisk Komité, senest 30 dage efter afgørelsen er modtaget. National Videnskabsetisk Komité kan, af hensyn til sikring af forsøgspersonernes rettigheder, behandle elementer af projektet, som ikke er omfattet af selve klagen.

© 2023 Todsen T et al. *JAMA Network Open*.

Klagen skal indbringes elektronisk og ved brug af digital signatur og kryptering, hvis protokollen indeholder fortrolige oplysninger. Dette kan ske på adressen: dketik@dketik.dk.

Klagen skal begrundes og være vedlagt kopi af Den Regionale Videnskabsetiske Komités afgørelse samt de sagsakter, som Den Regionale Videnskabsetiske Komité har truffet afgørelse på grundlag af.

NB: Der må ikke foretages ændringer i dokumenterne, som har været til behandling i komiteen, da sagen ellers vil blive sendt retur til komiteen.

Databeskyttelse - fortegnelseskrav
Du skal være opmærksom på, at du kan være forpligtet til at få forskningsprojektet fortegnet.
Er du forsker ansat i Region Hovedstaden, gør du dette ved at rette henvendelse til Videnscenter for Dataanmeldelser i Region Hovedstaden, som er den regionale enhed, der administrerer forskningsfortegnelsen. Du kan læse mere om fortegnelsen og finde kontaktoplysninger på videnscenterets hjemmeside.
Er du ikke ansat i Region Hovedstaden, kan du orientere dig om fortegnelseskravet i Vejledning om fortegnelse på Datatilsynets hjemmeside.

Med venlig hilsen

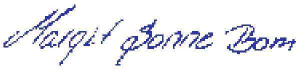

Margit Sonne Bom

Cand.jur.

© 2023 Todsen T et al. *JAMA Network Open*.

eFigure 1 Flowchart of the study design

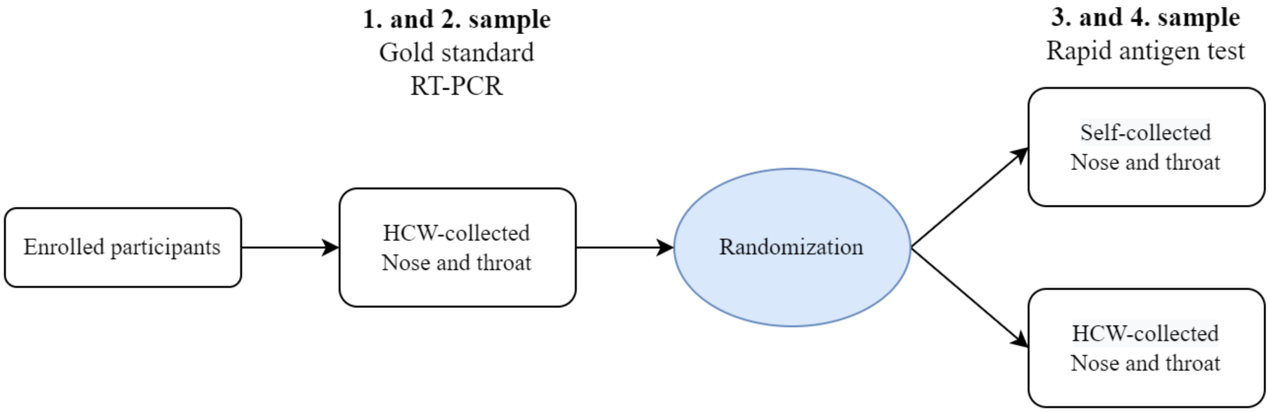

Abbreviations: RT-PCR: Reverse Transcriptase Polymerase Chain Reaction; HCW: Health care worker

© 2023 Todsen T et al. *JAMA Network Open*.

eFigure 2 Specificity of rapid antigen testing.

Specificity of self-collected and healthcare collected nasal and throat swabs for rapid antigen testing.

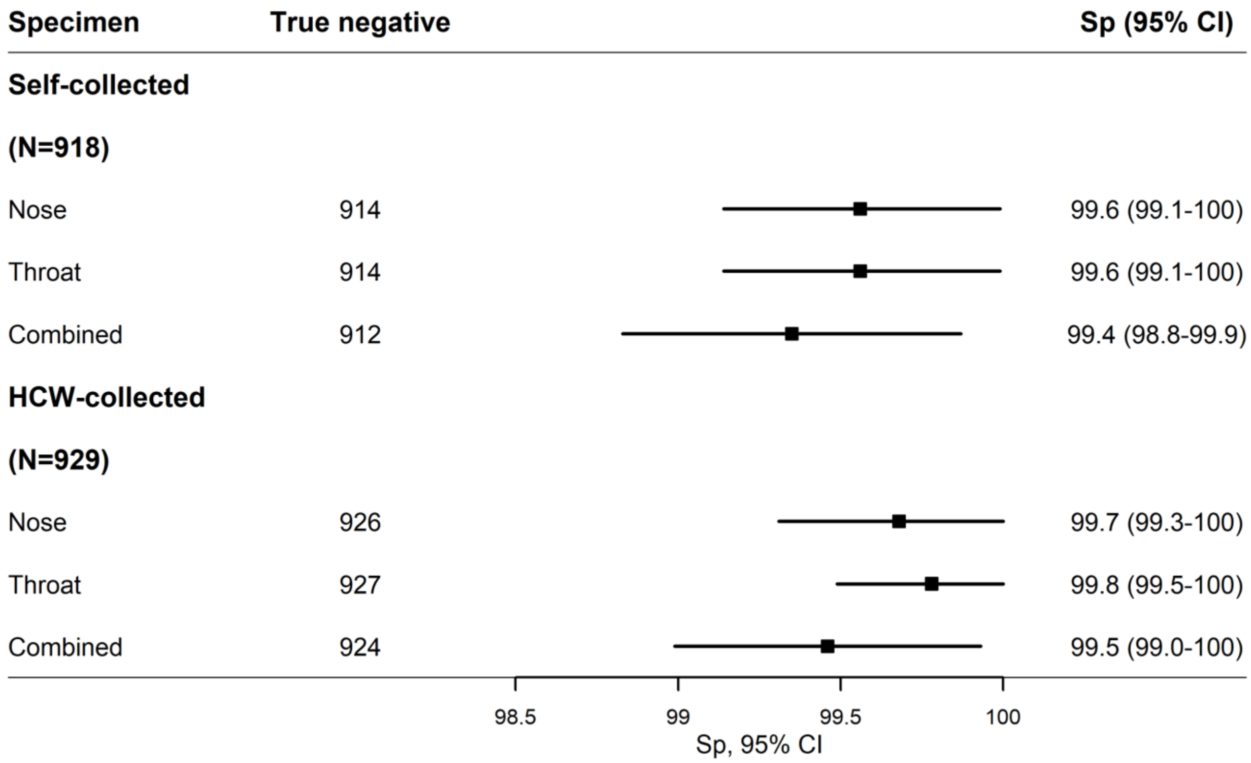

© 2023 Todsen T et al. *JAMA Network Open*.

eFigure 3 Forest plot of sensitivity with an intention-to-treat approach

Forest plot with sensitivity calculated with an intention-to-treat approach of self-collected and healthcare-collected nasal and throat swabs for rapid antigen testing.

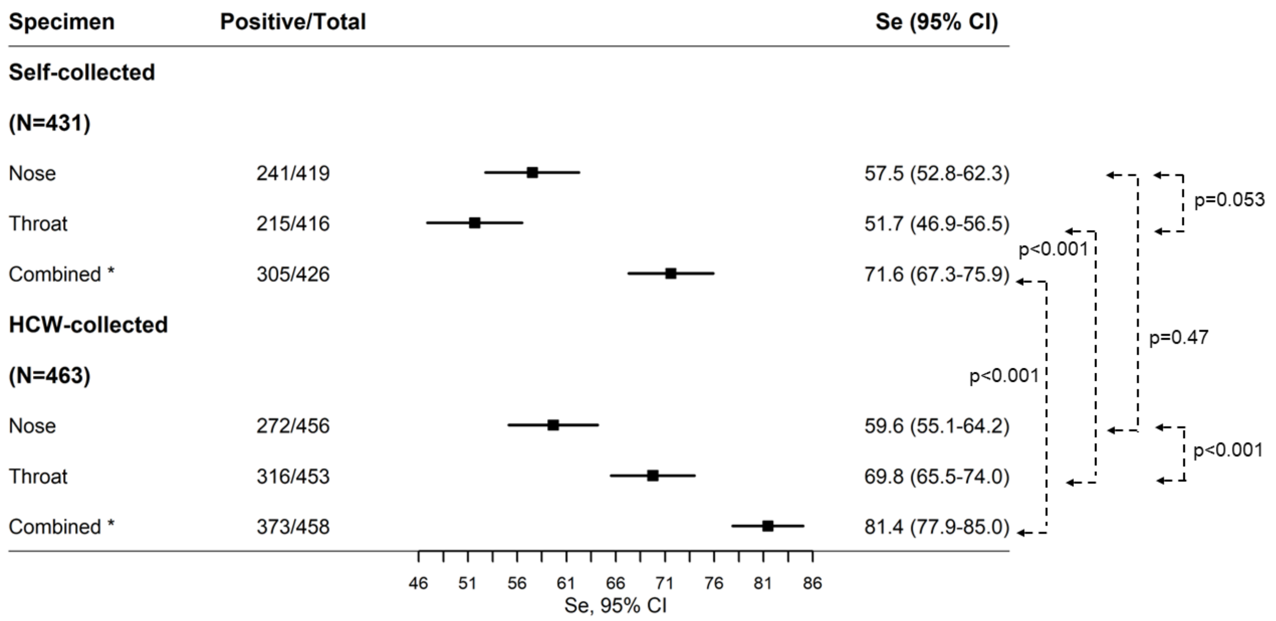

*Nasal and throat swabs were collected from the same participant but analyzed with individual rapid antigen testing devices, and detection rates were estimated based on the combined results from two individual test results.

© 2023 Todsen T et al. *JAMA Network Open*.

eFigure 4 Forest plot with sensitivity of rapid antigen testing stratified based on symptoms

Forest plot with sensitivity of self-collected and healthcare-collected nasal and throat swabs for rapid antigen testing stratified based on symptoms

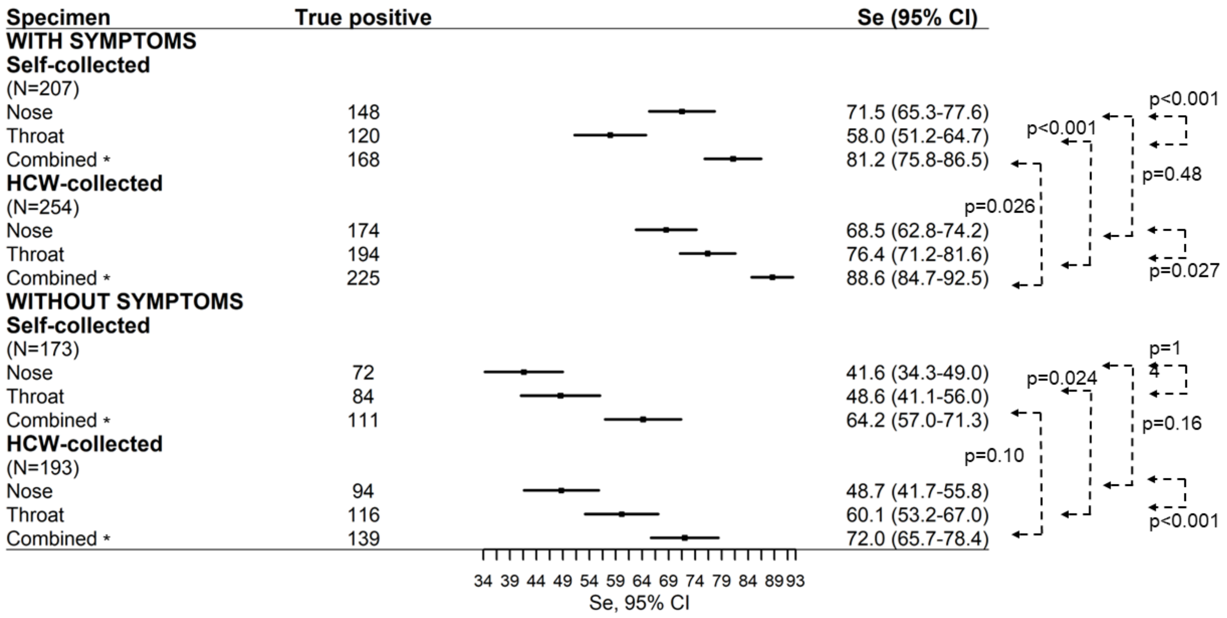

*Nasal and throat swabs were collected from the same participant but analyzed with individual rapid antigen testing devices, and detection rates were estimated based on the combined results from two individual test results.

© 2023 Todsen T et al. *JAMA Network Open*.

eFigure 5 Box plot with Ct values combined with the correlated rapid antigen results

Boxplot with Ct values for self-collected and healthcare collected (HCW) nasal and throat swabs, respectively, combined with the result of the correlated rapid antigen result (blue: positive, red: negative) for each test, only swabs analyzed at the Technical University of Denmark (DTU)

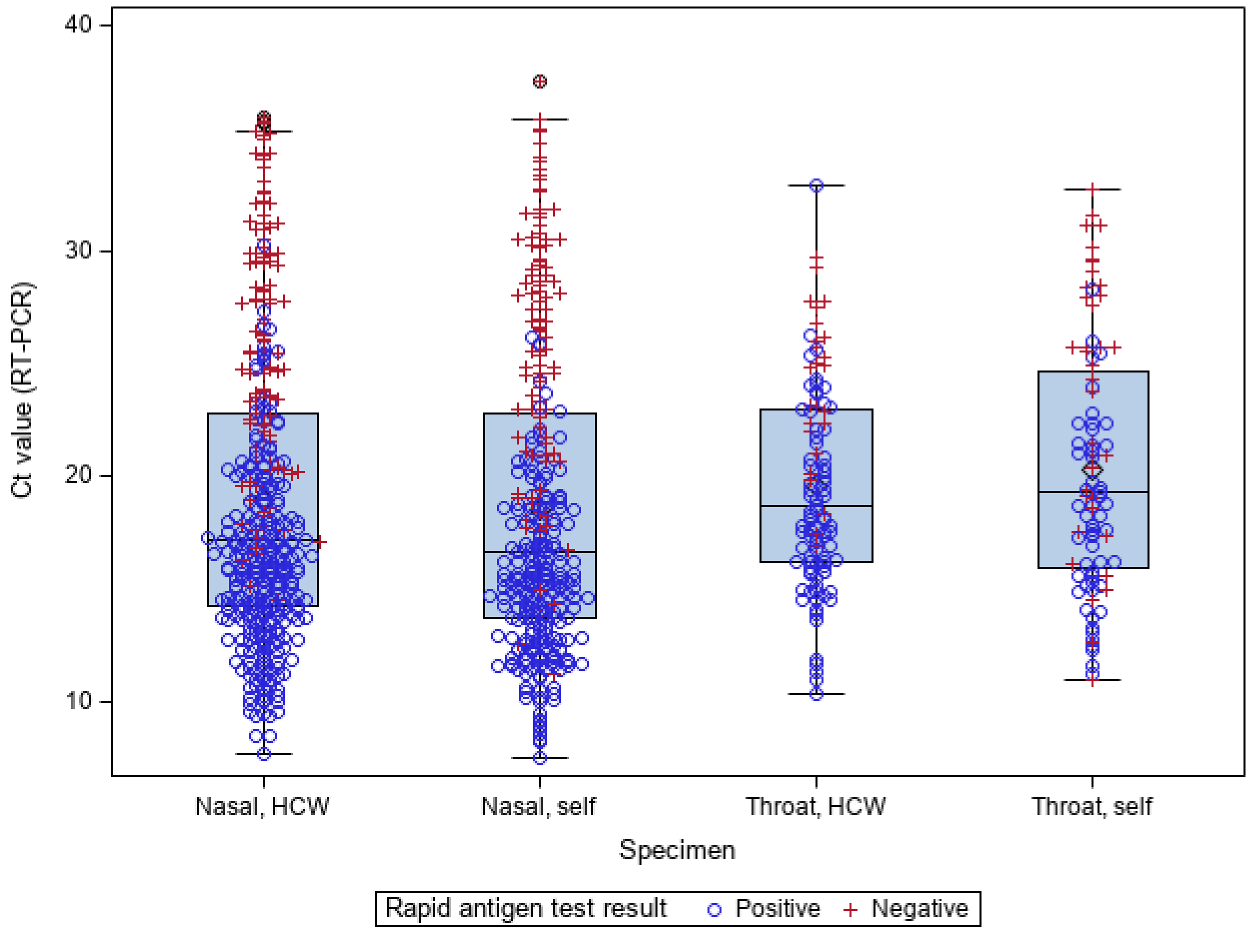

© 2023 Todsen T et al. *JAMA Network Open*.

eTable 1 Demographics and clinical characteristics of the enrolled and excluded participants

Demographics and clinical characteristics of the enrolled and excluded participants, N=2,941. Values are numbers and percentages (N, %) unless stated otherwise.

	All participants	Included	Excluded	p-value
Total	2,941 (100.0)	2,674 (90.9)	267 (9.1)	-
Sex				
Female	1,670 (56.8)	1535 (57.4)	135 (50.6)	0.032
Male	1,271 (43.2)	1139 (42.6)	132 (49.4)	
Age, median (Q_1-Q_3)	40 (28-55)	40 (28-55)	47 (30-60)	<0.001
Symptoms present				
Yes	1,166 (39.6)	1074 (40.2)	92 (34,5)	0.070
No	1,775 (60.4)	1600 (59.8)	175 (65.5)	
Vaccination status				
Vaccinated	2733 (94.2)	2484 (94.2)	249 (94.3)	0.96
Non-vaccinated	167 (5.8)	152 (5.8)	15 (5.7)	
Unknown	41	38	3	
Reason for testing				
COVID-19 like symptoms	1166 (39.6)	1074 (40.2)	92 (34.5)	0.25
Contact with infected person	919 (31.3)	833 (31.2)	86 (32.2)	
Regular testing	706 (24.0)	633 (23.6)	73 (27.3)	
Other	150 (5.1)	134 (5.0)	16 (6.0)	
Days since first symptoms[1]				
1 day				
2-3 days	409 (35.2)	379 (35.4)	30 (32.6)	0.79
4-6 days	573 (49.3)	527 (49.2)	46 (50.0)	
Missing	181 (15.5)	165 (15.4)	16 (17.4)	
	3	3	0	
Test center				
Copenhagen Airport	2617 (89.0)	2369 (88.6)	248 (92.9)	0.035
Valby	324 (11.0)	305 (11.4)	19 (7.1)	
PCR test results (pooled)				
Positive	894 (31.0)	827 (30.9)	67 (31.3)	0.91
Negative	1994 (69.0)	1847 (69.1)	147 (68.7)	
Missing	53	0	53	
Ag test results (nose)				
Positive	534 (18.8)	495 (18.5)	39 (24.1)	0.080
Negative	2302 (81.2)	2179 (81.5)	123 (75.9)	
Missing	105	0	105	
Ag test results (throat)				
Positive	548 (19.4)	520 (19.5)	28 (18.8)	0.84
Negative	2275 (80.6)	2154 (80.5)	121 (81.2)	
Missing	118	0	118	

© 2023 Todsen T et al. *JAMA Network Open*.

Abbreviations. Ag: Antigen; HCW: Healthcare worker.

[1]Among individuals with symptoms

© 2023 Todsen T et al. *JAMA Network Open*.

eTable 2 RT-PCR result for nasal and throat specimens

Comparison of RT-PCR performance by anatomical site of sampling

RT-PCR		Nasal		
		Positive	Negative	Total
Throat	Positive	668	111	779
	Negative	48	1,847	1,895
	Total	716	1,958	2,674

Abbreviations. PCR: polymerase chain reaction

© 2023 Todsen T et al. *JAMA Network Open*.

eTable 3 SARS-CoV-2 variants of concern

Subtype analysis of RT-PCR samples, N=827 PCR positive.

Analysis	**No. of cases (%)**	**Subtype**	**No. of cases (%)**	**% of successful analyzes**
Successful subtype analysis	286 (34.6)			
		BA.1	3 (0.36)	1.05
		BA.1.1	4 (0.48)	1.40
		BA.1.1.14	1 (0.12)	0.35
		BA.1.1.18	1 (0.12)	0.35
		BA.2	191 (23.1)	66.8
		BA.2.14	1 (0.12)	0.35
		BA.2.25	1 (0.12)	0.35
		BA.2.3	1 (0.12)	0.35
		BA.2.34	3 (0.36)	1.05
		BA.2.45	1 (0.12)	0.35
		BA.2.47	2 (0.24)	0.70
		BA.2.7	2 (0.24)	0.70
		BA.2.9	75 (9.1)	26.2
Failed analysis	30 (3.6)			
Missing specimen	511 (61.8)			

Sensitivity of self-collected and healthcare collected nasal and throat swabs for rapid antigen testing for two main subtypes.

Subtype	Randomization	N	Sampling	True positive	Se (95% CI)	p-value
BA.2	Self-collected	97	Nose	60	61.9 (51.4-71.5)	0.88
			Throat	59	60.8 (50.4-70.6)	
	HCW collected	94	Nose	60	63.8 (53.3-73.5)	0.26
			Throat	67	71.3 (61.0-89.1)	
BA.2.9	Self-collected	33	Nose	26	78.8 (61.1-91.0)	0.17
			Throat	21	63.6 (45.1-79.6)	
	HCW collected	42	Nose	27	64.3 (48.0-78.5)	NA
			Throat	36	85.7 (71.5-94-6)	

© 2023 Todsen T et al. *JAMA Network Open*.

eTable 4 Comparison of Rapid Antigen testing results from different sites with RT-PCR

HCW-COLLECTED SWABS FOR RAPID ANTIGEN TESTING

RT-PCR / Rapid Antigen Test	Positive	Negative	Total
**NASAL**			
Positive	268	3	271
Negative	179	926	1,105
Total	447	929	1,376
**THROAT**			
Positive	310	2	312
Negative	137	927	1,064
Total	447	929	1,376
**COMBINED**			
Positive	364	5	369
Negative	83	924	1,007
Total	447	929	1,376

SELF-COLLECTED SWABS FOR RAPID ANTIGEN TESTING

© 2023 Todsen T et al. *JAMA Network Open*.

RT-PCR / Rapid Antigen Test	Positive	Negative	Total
**NASAL**			
Positive	220	4	224
Negative	160	914	1,074
Total	380	918	1,298
**THROAT**			
Positive	204	4	208
Negative	176	914	1,090
Total	380	918	1,298
**COMBINED**			
Positive	279	6	285
Negative	101	912	1,013
Total	380	918	1,298

© 2023 Todsen T et al. *JAMA Network Open*.

eTable 5 Diagnostic accuracy calculated with an intention-to-treat approach of rapid diagnostic antigen tests

Diagnostic accuracy of rapid diagnostic antigen tests (sensitivity, specificity, positive predictive value, negative predictive value) of the enrolled participants based on randomization (self- or healthcare worker-collected). Inconclusive rapid diagnostic antigen test is defined as negative, N=2,941. Values are percentages (%) and 95% confidence interval.

	Rapid diagnostic antigen test					
	Self-collected (N=1,467)			HCW-collected (N=1,474)		
	Nose	Throat	Nose + throat	Nose	Throat	Nose + throat
Sensitivity	57.5 (52.8-62.3)	51.7 (46.9-56.5)	71.6 (67.3-75.9)	59.6 (55.1-64.2)	69.8 (65.5-74.0)	81.4 (77.9-85)
Specificity	99.4 (98.9-99.9)	99.6 (99.2-100)	99.2 (98.6-99.8)	99.7 (99.3-100)	99.8 (99.5-100)	99.5 (99-99.9)
PPV	97.6 (95.7-99.5)	98.2 (96.4-99.9)	97.4 (95.7-99.2)	98.9 (97.7-100)	99.4 (98.5-100)	98.7 (97.5-99.8)
NPV	84.6 (82.5-86.7)	83.0 (80.9-85.1)	89.0 (87.2-90.9)	84.0 (81.9-86.1)	87.6 (85.6-89.5)	91.9 (90.3-93.6)

Invalid Ag tests were defined as negative test results.

Abbreviations. HCW: Healthcare worker; PPV: Positive predictive value; NPV: Negative predictive value.

© 2023 Todsen T et al. *JAMA Network Open*.

eTable 6 Subgroup analysis comparing RT-PCR and Rapid diagnostic antigen test results between the 2 molecular laboratories.

Distribution of molecular test between different molecular laboratories, N (%) per specimen:

Specimen	PENTABASE (DTU)	COBAS (Rigshospitalet)	PENTABASE (Rigshospitalet)
Nasal	2,674 (100%)	0	0
Throat	757 (28.3%)	1,891 (70.7%)	26 (1.0%)

Diagnostic accuracy of rapid diagnostic antigen tests (sensitivity, specificity, positive predictive value, negative predictive value) of the enrolled participants based on randomization (self- or healthcare worker-collected) with RT-PCR testing at DTU, N=757. Values are percentages (%) and 95% confidence interval.

	Rapid diagnostic antigen test					
	Self-collected (N=370)			HCW-collected (N=387)		
	Nose	Throat	Nose + throat	Nose	Throat	Nose + throat
Sensitivity	54.6 (44.7-64.6)	55.7 (45.8-65.6)	69.1 (59.9-78.3)	53.9 (44.8-62.9)	71.8 (63.6-80.0)	80.3 (73.1-87.5)
Specificity	99.6 (98.9-100)	99.6 (98.9-100)	99.6 (98.9-100)	99.6 (98.9-100)	100 (100-100)	99.6 (98.9-100)
PPV	98.2 (94.6-100)	98.2 (94.7-100)	98.5 (95.7-100)	98.4 (95.4-100)	100 (100-100)	99.0 (96.9-100)
NPV	86.1 (82.3-89.9)	86.4 (82.6-90.1)	90.1 (86.7-93.4)	83.3 (79.2-87.4)	89.1 (85.6-92.6)	92.1 (89.0-95.2)

Missing and invalid Ag tests were excluded from these calculations.

Abbreviations. HCW: Healthcare worker; PPV: Positive predictive value; NPV: Negative predictive value.

© 2023 Todsen T et al. *JAMA Network Open*.

eTable 7 Rapid diagnostic antigen test results stratified by symptoms

Diagnostic accuracy of rapid diagnostic antigen tests (sensitivity, specificity, positive predictive value, negative predictive value) of the enrolled participants based on randomization (self- or healthcare worker-collected), N=2,674 stratified by symptoms (with, without). Values are percentages (%) and 95% confidence interval.

	Rapid diagnostic antigen test					
	Self-collected (N=1,298)			HCW-collected (N=1,376)		
	Nose	Throat	Nose + throat	Nose	Throat	Nose + throat
**Without symptoms**						
Sensitivity	41.6 (34.3-49.0)	48.6 (41.1-56.0)	64.2 (57.0-71.3)	48.7 (41.7-55.8)	60.1 (53.2-67.0)	72.0 (65.7-78.4)
Specificity	99.7 (99.2-100)	99.8 (99.5-100)	99.7 (99.2-100)	99.8 (99.5-100)	99.8 (99.5-100)	99.7 (99.3-100)
PPV	97.3 (93.6-100)	98.8 (96.5-100)	98.2 (95.8-100)	98.9 (96.9-100)	99.1 (97.5-100)	98.6 (96.6-100)
NPV	85.3 (82.7-88.0)	86.9 (84.3-89.4)	90.5 (88.2-92.7)	86.7 (84.2-89.1)	89.3 (87.0-91.6)	92.2 (90.3-94.2)
**With symptoms**						
Sensitivity	71.5 (65.3-77.6)	58.0 (51.2-64.7)	81.2 (75.8-86.5)	68.5 (62.8-74.2)	76.4 (71.2-81.6)	88.6 (84.7-92.5)
Specificity	99.4 (98.5-100)	99.1 (98.1-100)	98.8 (97.6-100)	99.3 (98.3-100)	99.6 (99.0-100)	98.9 (97.8-100)
PPV	98.7 (96.8-100)	97.6 (94.8-100)	97.7 (95.4-99.9)	98.9 (97.3-100)	99.5 (98.5-100)	98.7 (97.2-100)
NPV	84.7 (81.1-88.3)	78.9 (74.9-82.8)	89.3 (86.1-92.4)	78.0 (73.7-82.2)	82.6 (78.5-86.6)	90.7 (87.4-93.9)
	**Participants with symptoms divided by length.**					
**With symptoms, 1 day**						
Sensitivity	70.5 (60.4-80.6)	65.4 (54.8-75.9)	84.6 (76.6-92.6)	57.8 (47.6-68.0)	81.1 (73.0-89.2)	90.0 (83.8-96.2)
Specificity	99.1 (97.3-100)	99.1 (97.3-100)	99.1 (97.3-100)	99.0 (97.1-100)	100 (100-100)	99.0 (97.1-100)
PPV	98.2 (94.7-100)	98.1 (94.3-100)	98.5 (95.6-100)	98.1 (94.5-100)	100 (100-100)	98.8 (96.4-100)
NPV	82.6 (76.1-89.0)	80.1 (73.4-86.9)	90.1 (84.8-95.4)	72.5 (65-79.9)	85.6 (79.3-91.9)	91.7 (86.6-96.9)
**With symptoms, >1 day**						
Sensitivity	71.9 (64.1-79.7)	53.1 (44.5-61.8)	78.9 (71.8-86.0)	74.8 (68.2-81.5)	73.6 (66.9-80.4)	87.7 (82.7-92.8)
Specificity	99.5 (98.6-100)	99.1 (97.8-100)	98.6 (97.1-100)	99.5 (98.4-100)	99.5 (98.4-100)	98.9 (97.4-100)

© 2023 Todsen T et al. *JAMA Network Open*.

PPV	98.9 (96.8-100)	97.1 (93.2-100)	97.1 (93.9-100)	99.2 (97.6-100)	99.2 (97.6-100)	98.6 (96.7-100)
NPV	85.7 (81.4-90.0)	78.2 (73.3-83.1)	88.8 (84.8-92.8)	81.7 (76.6-86.8)	81.0 (75.9-86.1)	90.1 (86.0-94.2)

Missing and invalid Ag tests were excluded from these calculations.

Abbreviations. HCW: Healthcare worker; PPV: Positive predictive value; NPV: Negative predictive value.

© 2023 Todsen T et al. *JAMA Network Open*.

References

1. Charlton CL BE, Ginocchio CC, Hatchette TF, Jerris RC, Li Y, Loeffelholz M, McCarter YS, Miller MB, Novak-Weekley S, Schuetz AN, Tang Y-W, Widen R, Drews SJ. Practical Guidance for Clinical Microbiology Laboratories: Viruses Causing Acute Respiratory Tract Infections. Clinical Microbiology Reviews. 2018;32:e00042-18.
2. Jorgensen TS, Pedersen MS, Blin K, Kuntke F, Salling HK, Marvig RL, et al. SpikeSeq: A rapid, cost efficient and simple method to identify SARS-CoV-2 variants of concern by Sanger sequencing part of the spike protein gene. J Virol Methods. 2023;312:114648.

© 2023 Todsen T et al. *JAMA Network Open*.
