# Supplementary material for: COVID-19 Rapid Antigen Tests With Self-Collected vs Health Care Worker–Collected Nasal and Throat Swab Specimens: A Randomized Clinical Trial
Source: JAMA Netw Open. 2023 Dec 6;6(12):e2344295. doi: 10.1001/jamanetworkopen.2023.44295 (PMC10701611; doi:10.1001/jamanetworkopen.2023.44295)
Supplement: Supplement 3. — Data Sharing Statement [file jamanetwopen-e2344295-s003.pdf]

# Data Sharing Statement

Todsen. COVID-19 Rapid Antigen Tests With Self-Collected vs Health Care Worker–Collected Nasal and Throat Swab Specimens. *JAMA Netw Open*. Published December 06, 2023. doi:10.1001/jamanetworkopen.2023.44295

**Data**
**Data available:** Yes
**Data types:** Deidentified participant data
**How to access data:** Data will be available on web site (https://www.urt-sample.com/)
**When available:** With publication

**Supporting Documents**
**Document types:** Statistical/analytic code
**How to access documents:** Statistical code will be available on web site (https://www.urt-sample.com/)
**When available:** With publication

**Additional Information**
**Who can access the data:** anyone requesting the data
**Types of analyses:** or any purpose
**Mechanisms of data availability:** without investigator support
